# Genome-wide identification and expression analysis of the *CesA/Csls* gene family in *Eucalyptus Grandis*

**DOI:** 10.3389/fpls.2025.1624134

**Published:** 2025-10-13

**Authors:** Rui An, Yingtong Huang, Chunxia Lei, Ai-Min Wu, Chunjie Fan, Jianmei Long

**Affiliations:** ^1^ Guangdong Key Laboratory for Innovative Development and Utilization of Forest Plant Germplasm, College of Forestry and Landscape Architectures, South China Agricultural University, Guangzhou, China; ^2^ State Key Laboratory of Tree Genetics and Breeding, Key Laboratory of State Forestry and Grassland Administration on Tropical Forestry, Research Institute of Tropical Forestry, Chinese Academy of Forestry, Guangzhou, China

**Keywords:** glycosyltransferase, CesA/Csls gene family, cell wall, *Eucalyptus grandis*, expression analysis

## Abstract

**Introduction:**

The cellulose synthase gene superfamily is composed of two major gene families: cellulose synthase (*CesA*) and cellulose synthase-like (*Csl*). These genes play a crucial role in the synthesis of cellulose and hemicellulose within the plant cell wall, and are essential for controlling plant growth and development. However, the *CesA/Csls* gene family has not been previously reported in *Eucalyptus grandis*.

**Methods:**

In this study, bioinformatics methods were employed to identify the CesA/Csls gene family inE. grandisand to analyze the potential functions of its members in cell wall formation.

**Results:**

The results revealed that there were 62 *CesA/Csls* family members inE. grandis, which were classified into seven subfamilies (*CesA, CslA, CslB, CslC, CslD, CslE, CslG*) based on evolutionary tree analysis. Promoter regions of these genes contained various cis-acting elements, with light-responsive elements being the most abundant. Gene expression pattern analysis showed that *EgCesA4, EgCesA7*, and *EgCesA8* were highly expressed in the xylem, suggesting their primary association with cellulose synthesis during secondary wall thickening (lignification).

**Discussion:**

Overall, the analysis of the *EgCesA/Csls* gene family provides a valuable reference for understanding cellulose synthesis in the cell wall, genetic improvement, and biotechnological applications.

## 1 Introduction

The cell wall, a key structural component of plant cells, was essential for regulating cell morphology, driving growth and development, and enabling cell expansion (Persson et al., 2007a). Cellulose, hemicellulose and pectin were the principal components of primary cell walls, while cellulose, hemicellulose and lignin were the principal components of secondary cell walls (Burton et al., 2010). It was evident that cellulose performed a pivotal function in processes such as cell growth, differentiation and signaling. In addition, hemicellulose had been observed to interact closely with cellulose microfibrils through hydrogen bonding (Welker et al., 2015).

Glycosyltransferases (GTs) are enzymes that catalyze the transfer of an activated sugar group to an acceptor substrate, which may consist of polysaccharides, peptides, lipids, or small molecules (Zabotina et al., 2021). Cellulose, hemicellulose, pectin, and lignin are the major polysaccharides in plants (Li et al., 2020). The cellulose synthase gene superfamily is a group of type-two glycosyltransferases (GT2), which is the largest family within GTs (Aspeborg et al., 2005). This superfamily could be further divided into two subfamilies: cellulose synthase (*CesA*) and nine cellulose synthase-like (*Csl*) subfamilies (*CslA/B/C/D/E/F/G/H/J*) (Yin et al., 2009, 2014). Cellulose was primarily synthesized by *CesA* (cellulose synthase), while some hemicelluloses were synthesized with the involvement of *Csl*s (cellulose synthase-like proteins) (Yin et al., 2014). A new *CslM* subfamily had been identified in other flowering plants (Farrokhi et al., 2006; Little et al., 2018). Available studies had shown that the *Csl*A gene was involved in the biosynthesis of the mannan and glucomannan backbone (Liepman et al., 2005; Goubet et al., 2009). The *CslC* genes, on the other hand, were responsible for the biosynthesis of the β-1,4-glucan backbone for xyloglucan synthesis (Cocuron et al., 2007). The *Cs1D* gene might be involved in the synthesis of cellulose or mannan in tip-growing cells (Park et al., 2011; Yin et al., 2011; Yang et al., 2020). In contrast, the *CslF*, *CslH*, and *CslJ* subfamilies catalyzed the biosynthesis of (1,3;1,4)-β-glucan, which were polymers commonly referred to as mixed ligated glucan (MLG) (Doblin et al., 2009; Vega-Sánchez et al., 2012; Little et al., 2019; Lou et al., 2022). However, the specific functions of the other four *Csl* subfamily members (*CslB*, *CslE*, *CslG* and *CslM*) remained to be elucidated.

The *CesA/Csl* gene families play a pivotal role in plant cell wall biosynthesis by mediating the synthesis of both cellulose and hemicellulose polysaccharides (McFarlane et al., 2014). In *A. thaliana*, *AtCesA1*, *AtCesA3* and *CesA6-like* genes (*AtCesA*2/5/6/9) had been reported to be involved in primary wall cellulose synthesis, whereas the *AtCesA4*, *AtCesA7* and *AtCesA8* genes were responsible for secondary wall cellulose synthesis (Sod et al., 2023). Multiple *CesA* proteins assembled into the cellulose synthase complex *CesA* complexe (CSC), which was responsible for cellulose synthesis (Zhang et al., 2021). *Csl*s, a member of the cellulose synthase superfamily, shared high sequence similarity with the *CesA* gene family.

*CesA/Csl* played a crucial role in plant growth and development (Polko and Kieber, 2019). For instance, the impairment of rice OsCSLD4 function resulted in a reduction of grain width and weight, whereas the overexpression of OsCSLD4 led to an increase in both grain width and weight (Zhao et al., 2022). The knockdown of *HvCslF3* from barley through RNA interference resulted in a decrease in root growth rate, which was associated with a reduced elongation zone and a marked reduction in the root system (Lou et al., 2022). The PbrMADS52-PbrCSLD5 signaling pathway resulted in an elevated cellulose content within the cell wall of pear pollen tubes, consequently hindering the growth of the pollen tubes in *Pyrus bretschneideri* (Li et al., 2022). Likewise, the single mutants *csld1* and *csld4*, as well as the double mutant *csld1*/*csld4* in Arabidopsis, exhibited highly irregular pollen tube growth. This abnormality led to a substantial decrease in cellulose accumulation within the pollen tube wall and a pronounced disorganization of the pollen tube wall layers, ultimately impairing the genetic transmission of male gametophytes (Wang et al., 2011). Cellulose constitutes the primary component of secondary cell walls. In addition, *CesA/Csls* were also involved in stress response in plants. For instance, in rice, high temperature activates the kinase *OsCDPK24/28*, which phosphorylates *OsHSFA4d* at S146. This phosphorylation event subsequently leads to the differential regulation of *HSP101* and *CslF6* gene expression (Fang et al., 2025). Parallel studies examining gene expression changes in orchid stems under abiotic stress revealed distinct roles for *Csl* subfamilies: *CslA* appears to be a key subfamily in drought stress response across orchids of different life forms, whereas *CslD* may be particularly important for epiphytic and saprophytic orchids in adapting to freezing stress (Wang et al., 2022).

*E. grandis*, as one of the world’s three fast-growing tree species, has important economic, ecological and social values, and is widely used in paper, timber and other industrial fields. With the completion of genome sequencing of *E. grandis* (Myburg et al., 2014), a solid foundation for molecular biology research and genetic improvement of *Eucalyptu*s has been provided. To date, the *CesA/Csl* gene family had been comprehensively characterized and studied in numerous plant species, including rice *Oryza sativa* (Ouyang et al., 2007), *Sorghum bicolor* (Paterson et al., 2009), *Gossypium hirsutum* (Li et al., 2017), *Linum usitatissimum* (Pydiura et al., 2015), *Populus trichocarpa* (Takata and Taniguchi, 2015) and *Arabidopsis thaliana* (Persson et al., 2007b). However, the *CesA/Csl* genes has not been identified in *E. grandis.* In view of the important roles of *CesA* and *Csl*s genes in plant growth and development, the present study systematically identified the *CesA* and *Csl*s family members in *E. grandis*, and analyzed their characteristics, including phylogenetic relationships, gene and protein structural characteristics, *cis*-acting elements and gene expression patterns. These results laid a theoretical foundation for in-depth study of the functions of *CesA/Csls* genes in cell wall synthesis during growth and development process in *E. grandis*.

## 2 Materials and methods

### 2.1 Identification and physicochemical characterization of *EgCesA/Csls* gene family members

The *Arabidopsis* AtCesA/Csls protein sequences were initially obtained by downloading from the TAIR database (http://www.arabidopsis.org), and used as query sequences to search the candidate CesA/Csls in *E. grandis* via the BlastP program, with a threshold set at E value of e-10. The *CesA/Csls* family feature domain (PE00535 and PF03552) vas used as a query sequence. Meanwhile, bidirectional BLAST alignment using TBtools software (v2.110, (Chen et al., 2020) and screening via the Hidden Markov Model (HMM) were conducted. In these two ways, the candidate EgCesA/Csls proteins were finally obtained. The basic physicochemical properties of EgCesA/Csls proteins, including amino acid length, theoretical isoelectric point, relative molecular mass, grand average of hydropathicity, instability index, and aliphatic index, were analyzed using the ProtParam tool in ExPASy (https://web.expasy.org/protparam/) (Li et al., 2024), and WoLF PSORT (https://wolfpsort.hgc.jp/) was used for subcellular localization prediction. The genome sequence and annotation information of *E. grandis* was obtained from the National Center for Biotechnology Information (NCBI) database with the accession number ASM1654582v1.

### 2.2 Phylogenetic tree construction of *EgCesA/Csls* family genes

To investigate the phylogenetic relationship of the *CesA/Csls* gene family, the amino acid sequences of *CesA/Csls* of three species were selected, including *Arabidopsis thaliana* (*A. thaliana*), *Populus trichocarpa* (*P. trichocarpa*) and *Physcomitrella patens* (*P. patens*) (**Supplementary Table 1**). Sequence alignment and similarity analysis were conducted on these four species using MEGA. Genomic annotation information for *P. patens* and *P. trichocarpa* was obtained from NCBI (https://www.ncbi.nlm.nih.gov/) with the accession number GCF_000002425.5 and GCF_000002775.5. The phylogenetic tree was built using the Maximum Likelihood (ML) method, with parameters set to UltraFast BootStrap, a Bootstrap number of 1000 (model: Auto, number of steps: Auto), and other parameters set to their default values. The phylogenetic tree was visualized and optimized using the Evolview website (https://www.evolgenius.info).

### 2.3 Analysis of *EgCesA/Csls* gene structure and conserved structural domains

The conserved domains of EgCesA/Csls proteins were predicted using CDD-Search program in NCBI (https://www.ncbi.nlm.nih.gov/cdd). Multiple Expectation Maximization for Motif Elicitation (MEME) (https://meme-suite.org/meme/index.html) was used to identify the conserved motifs of EgCesA/Csls proteins, with the number of maximum motifs set to 10 and *p*-value less than 1e-5. The exon-intron structures of the *EgCesA/Csls* genes were generated using TBtools (v2.110) based on their genome DNA sequence and coding sequence (CDS). Additionally, the gene structure conserved motifs and domains of EgCesA/Csls proteins were visualized by TBtools.

### 2.4 Chromosomal localization and collinearity analysis of the *EgCesA/Csls* gene family

The GFF file from the *E. grandis* genome was utilized to obtain chromosomal location information for members of the *EgCesA/Csls* gene family and to generate a distribution map of these genes on chromosomes by TBtools. The “One Density Profile” program was used, with the parameter “Bin Size” set to 1,000,000. To identify the gene duplication patterns, synteny analyses of the *CesA/Csls* genes in *E. grandis vs*. *A. thaliana* and *E. grandis vs*. *P. trichocarpa* were performed using the Dual Systeny Plot program of TBtools. To investigate the internal collinear relationships within the *E. grandis* genome, a synteny analysis was conducted across the entire genome utilizing the “One Step MCScanX” tool, with results visualized via the “Advanced Circos” module (Chen et al., 2020). For the MCScanX run, the analysis parameters were set as follows: BLASTP alignment was performed using 2 CPU threads, an E-value cutoff was set at 10^-10^, and the five highest-ranking BLAST matches were retained for subsequent synteny analysis.

### 2.5 Analysis of *cis-*acting elements in the *EgCesA/Csls* family promoter

We extracted 2000 bp upstream of the transcriptional start site (ATG) of *EgCesA/Csls* gene from *E. grandis* genome by using ‘GXF’ Sequence Extraction’ tool in TBtools-II software. Subsequently, these sequences were compared and analyzed against the online PlantCare database (https://bioinformatics.psb.ugent.be/webtools/plantcare/html/)to identify the *cis-*acting elements of this gene family. Finally, the retrieved *cis-*acting elements were integrated with the phylogenetic tree of *E. grandis* and visualized using the “Basic BioSequence View” tool in TBtools-II. To predict the transcription factor binding site in the promoter of *EgCesA/Csls*, the promoter sequences of *EgCesA/Csls* gene were uploaded to PlantRegMap (http://plantregmap.gao-lab.org/), with the parameter of *p*-value less than 1e^-7^.

### 2.6 Expression patterns of the *EgCesA/Csls* gene family

To examine the tissue-specific expression patterns of *E. grandis CesA/Csls* family genes, transcriptome data were used from our previous study (Fan et al., 2024). The raw sequencing reads have been archived in the Genome Sequence Archive (GSA) at the National Genomics Data Center under the accession number PRJCA002468. Plant materials were derived from *in vitro*-propagated shoots with established root systems, cultivated under controlled conditions at the Research Institution of Tropical Forestry, Chinese Academy of Forestry (Guangzhou, China; coordinates: E 113.385°, N 23.191°). From six-month-old greenhouse-grown shoots at the same institution, multiple tissue types were harvested: roots, mature leaves, young leaves, xylem, and phloem. Additionally, sequential stem internodes (1st, 3rd, 5th, 7th, 9th, and 11th) were collected from these shoots. Tissues including xylem, phloem, and cambium were also sampled from six-year-old trees located at the Zhenshan nursery (Sihui, Zhaoqing, Guangdong, China; coordinates: E 112.673°, N 23.330°).

To induce nutrient deficiency, two-month-old plants were exposed to boron-deficient or phosphorus-deficient regimes using half-strength Hoagland’s nutrient solution. The solution pH was maintained at 5.8 through adjustment with NaOH. Post-treatment, root tissues were gently dried, rapidly frozen in liquid nitrogen, and stored at -80°C. For investigations into hormonal responses and abiotic stress, shoots were propagated *in vitro* and subsequently grown in potted conditions within the greenhouse for two months. Prior to treatment, fully expanded young leaves (4–8 leaves per plant) situated immediately below the shoot apex were selected from plants reaching 25–35 cm in height. Hormonal elicitation involved foliar application of 100 μM salicylic acid (SA) or methyl jasmonate (MeJA). Leaf samples subjected to hormonal treatments or salinity stress (imposed via irrigation with 200 mM NaCl) were collected at defined intervals post-application: 0, 1, 6, 24, and 168 hours. For each organ type and treatment condition, biological replicates consisted of pooled tissue from a minimum of three individual plants, with the entire experiment performed in triplicate.

Using the TBtools Heatmap function, transcriptome data from 6-month-old branches were logarithmically normalized using the formula “logbase(value+LogWith)” (where base = 2.0 and LogWith = 1.0) to minimize data dispersion. Subsequently, “Cluster Rows” was selected for hierarchical clustering. A heatmap depicting expression patterns was generated after hierarchical clustering. Additionally, using the TBtools Heatmap function, logarithmic scaling was chosen for logarithmic normalization of the transcriptome data. The tile shape was set to “circular,” and “scale size by area” was selected to create an initial expression heatmap. Furthermore, after logarithmic normalization, “row scaling” was applied to normalize the rows, thereby enhancing the clarity of differential expression in the heatmap. The heatmaps were then combined to comprehensively illustrate the expression patterns of the *EgCesA/Csls* gene family under abiotic stress and phytohormone treatments.

### 2.7 *EgCesA/Csls* protein structure prediction

Utilizing the Swiss-Model online tool (https://swissmodel.expasy.org/), the three-dimensional structures of the *EgCesA/Csls* family proteins were constructed through homology modeling. Additionally, Alphafold (https://alphafoldserver.com) was utilized to predict the structural configurations of the protein complexes formed by EgCesA4, EgCesA7, and EgCesA8, which were potentially implicated in the synthesis of secondary walls.

## 3 Results and analysis

### 3.1 Gene family identification and physicochemical analysis results

In this study, 62 *CesA/Csls* proteins were successfully identified through BLASTp alignment. Based on the nomenclature of this gene family in the model plant *A. thaliana*, we have assigned corresponding names to the genes in *E. grandis*. Among them, 21 cellulose synthases sequentially named as *EgCesA1a to EgCesA1e, EgCesA3a to EgCesA3c, EgCesA6a to EgCesA6i, EgCesA4, EgCesA7, EgCesA8, EgCesA10*; the remaining 41 belonged to four types of cellulose synthase-likes, named *EgCslB1* to *EgCslB4*, *EgCslC4a*, *EgCslC4c*, *EgCslC6*, *EgCslC12a*, *EgCslC12b*, *EgCslD1*, *EgCslD2, EgCslD4, EgCslD6a, EgCslD6b*, *EgCslE1a* to *EgCslE1k*, and *EgCslG1* to *EgCslG12* (**Table 1**). An analysis of the physicochemical properties of *EgCesA/Csls* indicated that the number of amino acids in these proteins ranged from 166 to 1138. Specifically, the protein encoded by the *EgCslD2* gene had the highest number of amino acids, reaching 1138, while the *EgCslE1j* had the lowest with only 166 amino acids. The isoelectric point analysis showed that 21 genes had isoelectric points below 7, while the remaining genes had isoelectric points above 7, indicating that the *EgCesA/Csls* gene family was predominantly composed of basic amino acids. The aliphatic index ranged from 74.36 (*EgCslG8*) to 118.16 (*EgCesA1c*), reflecting significant differences in the thermal stability of proteins within this family. In terms of hydrophilicity analysis, 40 *EgCesA/Csls* genes exhibited negative values, confirming their hydrophilic nature. Among them, *EgCslG8* had the highest overall average hydrophilicity (GRAVY) of -0.423, while *EgCesA1c* had the lowest GRAVY of 0.599 (**Table 1**), indicating that the overall hydrophilicity of this family was not prominent.

**Table 1:** The basic information of identified *EgCesA/Csls* gene family members.

Name	Sequence ID	Number of amino acids	Molecular weight	Theoretical pI	Instability index	Aliphatic index	Grand average of hydropathicity	Subcellular localization
EgCesA1a	Eucgr.C02801.1.v2.0	1085	122055.76	6.38	41.36	85.17	-0.239	plasma membrane
EgCesA1b	Eucgr.L02402.1.v2.0	590	66356.73	6.77	32.47	93.69	0.022	plasma membrane
EgCesA1c	Eucgr.C01147.1.v2.0	217	24409.43	6.50	16.17	118.16	0.599	plasma membrane
EgCesA1d	Eucgr.J01639.1.v2.0	505	57201.35	8.68	39.52	92.89	-0.044	plasma membrane
EgCesA1e	Eucgr.H00939.1.v2.0	569	64189.42	7.53	37.30	96.64	0.085	plasma membrane
EgCesA3a	Eucgr.A02372.1.v2.0	869	98210.09	8.47	40.76	90.08	-0.103	plasma membrane
EgCesA3b	Eucgr.G03380.2.v2.0	1080	121058.56	6.84	39.72	86.18	-0.186	plasma membrane
EgCesA3c	Eucgr.J01278.1.v2.0	1079	121000.81	7.01	40.58	86.08	-0.186	plasma membrane
EgCesA4	Eucgr.A01324.1.v2.0	924	104599.53	8.95	37.93	89.00	-0.100	plasma membrane
EgCesA6a	Eucgr.B01562.1.v2.0	888	100750.78	8.46	36.14	87.70	-0.131	plasma membrane
EgCesA6b	Eucgr.F03635.1.v2.0	1097	123599.32	6.93	38.87	84.77	-0.215	plasma membrane
EgCesA6c	Eucgr.B01532.1.v2.0	896	101496.72	8.37	36.63	89.00	-0.109	plasma membrane
EgCesA6d	Eucgr.B03971.1.v2.0	788	87777.96	8.75	45.72	83.67	-0.219	plasma membrane
EgCesA6e	Eucgr.F04216.1.v2.0	1092	123632.76	6.82	39.86	88.35	-0.187	plasma membrane
EgCesA6f	Eucgr.F04212.1.v2.0	1091	123536.87	7.63	41.14	88.27	-0.183	plasma membrane
EgCesA6g	Eucgr.H00646.1.v2.0	672	76211.99	8.64	34.53	92.98	-0.007	plasma membrane
EgCesA6h	Eucgr.H02200.1.v2.0	582	65499.77	9.15	35.58	95.12	0.057	plasma membrane
EgCesA6i	Eucgr.I00286.1.v2.0	1092	123273.49	6.31	40.36	84.41	-0.254	plasma membrane
EgCesA7	Eucgr.C00246.1.v2.0	1041	117640.43	6.31	41.83	81.29	-0.216	plasma membrane
EgCesA8	Eucgr.D00476.1.v2.0	978	109871.56	6.36	40.06	85.83	-0.079	plasma membrane
EgCesA10	Eucgr.C01769.1.v2.0	1082	121787.70	6.72	39.07	86.04	-0.218	plasma membrane
EgCslA2	Eucgr.J00420.1.v2.0	527	61028.68	9.13	37.60	99.09	0.172	plasma membrane
EgCslA9a	Eucgr.G02715.1.v2.0	482	55519.97	9.20	36.14	91.56	0.056	plasma membrane
EgCslA9b	Eucgr.A01558.1.v2.0	533	61016.64	9.21	34.87	98.91	0.158	plasma membrane
EgCslB1	Eucgr.K00778.1.v2.0	667	74333.49	6.26	40.60	86.54	-0.016	plasma membrane
EgCslB2	Eucgr.K00782.1.v2.0	816	92242.84	8.50	41.12	91.43	0.012	plasma membrane
EgCslB3	Eucgr.K00779.1.v2.0	754	84888.31	8.36	39.89	87.81	0.023	plasma membrane
EgCslB4	Eucgr.K00781.1.v2.0	479	53961.07	8.48	46.72	81.06	-0.248	nucleus
EgCslC4a	Eucgr.F04010.1.v2.0	648	74780.78	9.22	35.59	101.71	0.050	plasma membrane
EgCslC4b	Eucgr.I01833.1.v2.0	667	76610.75	8.78	37.25	103.22	0.139	plasma membrane
EgCslC4c	Eucgr.C02007.1.v2.0	694	79603.20	8.90	35.24	100.69	0.084	plasma membrane
EgCslC6	Eucgr.H00749.1.v2.0	572	64610.13	7.92	43.00	94.58	-0.080	plasma membrane
EgCslC12a	Eucgr.F02219.1.v2.0	709	80593.63	8.08	41.08	95.99	0.003	plasma membrane
EgCslC12b	Eucgr.F00101.1.v2.0	707	80298.26	8.69	42.00	96.01	-0.003	plasma membrane
EgCslD1	Eucgr.H00079.1.v2.0	1051	117936.95	8.07	46.61	81.17	-0.232	plasma membrane
EgCslD2	Eucgr.K00085.1.v2.0	1138	127549.43	7.24	43.09	83.44	-0.196	plasma membrane
EgCslD4	Eucgr.H05010.1.v2.0	1120	125713.61	6.05	43.76	78.73	-0.216	plasma membrane
EgCslD6a	Eucgr.D02228.1.v2.0	1116	126105.01	7.47	43.95	82.66	-0.204	plasma membrane
EgCslD6b	Eucgr.E00226.1.v2.0	1100	123978.72	6.44	39.36	82.82	-0.233	plasma membrane
EgCslE1a	Eucgr.F03681.1.v2.0	739	83721.60	6.69	52.14	87.74	-0.010	plasma membrane
EgCslE1b	Eucgr.E03851.1.v2.0	732	83520.95	6.27	46.12	84.33	-0.112	chloroplast
EgCslE1c	Eucgr.E03847.1.v2.0	479	54440.74	5.97	37.81	92.59	-0.037	chloroplast
EgCslE1d	Eucgr.L03518.1.v2.0	423	47868.29	8.40	39.15	86.41	-0.030	nucleus
EgCslE1e	Eucgr.E03849.1.v2.0	742	85231.45	8.33	46.52	86.47	-0.113	plasma membrane
EgCslE1f	Eucgr.H05074.1.v2.0	661	75821.69	7.78	45.41	83.65	0.026	plasma membrane
EgCslE1g	Eucgr.L01389.1.v2.0	445	51497.59	5.71	51.01	82.81	-0.332	cytosol
EgCslE1h	Eucgr.L01392.1.v2.0	263	30959.81	8.64	53.77	92.66	-0.126	cytosol
EgCslE1i	Eucgr.L01391.1.v2.0	227	26235.83	9.24	43.13	97.93	0.206	cytosol
EgCslE1j	Eucgr.L01388.1.v2.0	166	18511.83	8.34	31.94	105.78	0.466	plasma membrane
EgCslE1k	Eucgr.E03846.1.v2.0	655	74934.83	8.35	43.95	91.85	-0.012	plasma membrane
EgCslG1	Eucgr.D01765.1.v2.0	737	82569.62	7.85	43.91	93.07	0.085	plasma membrane
EgCslG2	Eucgr.D01766.1.v2.0	442	49111.14	8.78	38.78	92.44	0.183	plasma membrane
EgCslG3	Eucgr.D01768.1.v2.0	722	80278.80	8.38	42.03	93.10	0.094	plasma membrane
EgCslG4	Eucgr.H00189.1.v2.0	725	81799.38	6.51	45.24	83.77	-0.003	plasma membrane
EgCslG5	Eucgr.E00821.1.v2.0	745	83704.72	6.89	46.91	91.19	0.046	plasma membrane
EgCslG6	Eucgr.E00820.1.v2.0	624	70725.01	6.78	49.40	85.13	-0.116	plasma membrane
EgCslG7	Eucgr.E00819.1.v2.0	502	56388.56	8.70	46.59	93.75	0.200	plasma membrane
EgCslG8	Eucgr.H00185.1.v2.0	388	44672.30	8.46	49.00	74.36	-0.423	chloroplast
EgCslG9	Eucgr.H00187.1.v2.0	516	58938.10	6.00	41.75	83.66	-0.150	plasma membrane
EgCslG10	Eucgr.H00188.1.v2.0	705	80295.96	7.02	44.03	82.26	-0.083	plasma membrane
EgCslG11	Eucgr.L03024.1.v2.0	280	31473.13	7.51	35.73	99.89	0.378	plasma membrane
EgCslG12	Eucgr.H00186.1.v2.0	737	83886.50	8.46	43.18	86.49	-0.006	plasma membrane

### 3.2 Phylogenetic tree of the *EgCesA/Csls* gene family

**Figure 1:**
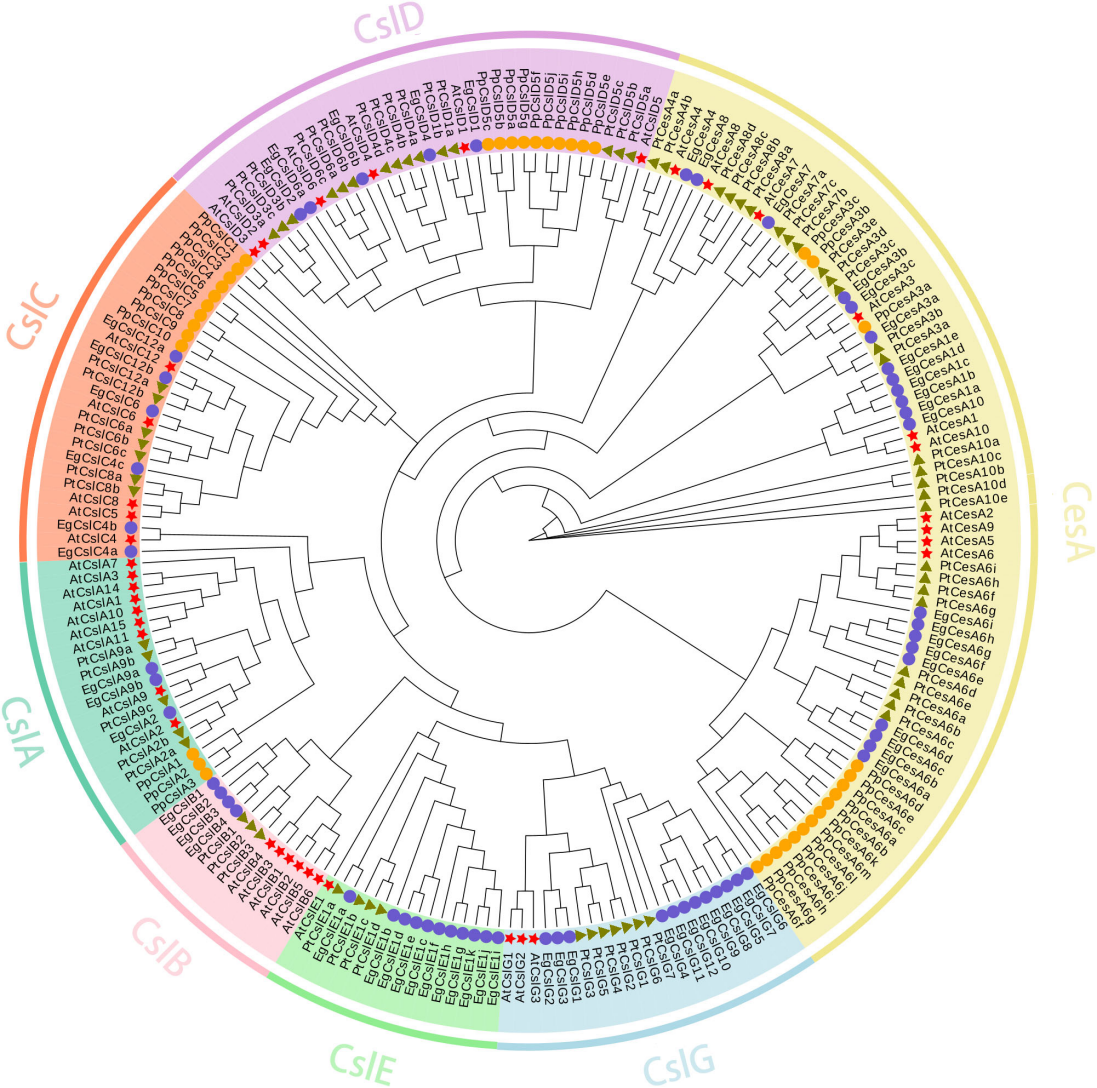
Phylogenetic analysis of *CesA/Csls* genes in *E. grandis*, *A. thaliana*, *P. trichocarpa*, and *P. patens*. Signs of different shapes represent *CesA/Csls* genes from *E. grandis* (blue round, Eg), *A. thaliana* (red star, AT), *P. trichocarpa* (green triangle, Pt), and *P. patens* (orange round, Pp).

To investigate the functions of *EgCesA/Csls*, a phylogenetic tree was constructed based on 210 protein sequences from *E. grandis*, *A. thaliana, P. trichocarpa, and P. patens* using TBtools-II software. The result revealed that whole *CesA/Csls* proteins could be classified into seven subfamilies, namely one *CesA* subfamily and six *Csl* subfamilies (*CslA, CslB, CslC, CslD, CslE, CslG*). The *EgCesA* subfamily contained 21 members, while the *EgCslA* subfamily consisted of 3 members; the *EgCslB* subfamily comprised 4 members, the *EgCslC* subfamily included 6 members, the *EgCslD* subfamily had 5 members, the *EgCslE* subfamily had 11 members, and the *EgCslG* subfamily contained 12 members. Additionally, the phylogenetic analysis indicated that the *EgCesA/Csls* genes were more closely related to those of *P. trichocarpa* compared to *A. thaliana* and *P. patens* (**Figure 1**).

### 3.3 Analysis of the gene structure and conserved domains of the *EgCesA/Csls* family

**Figure 2:**
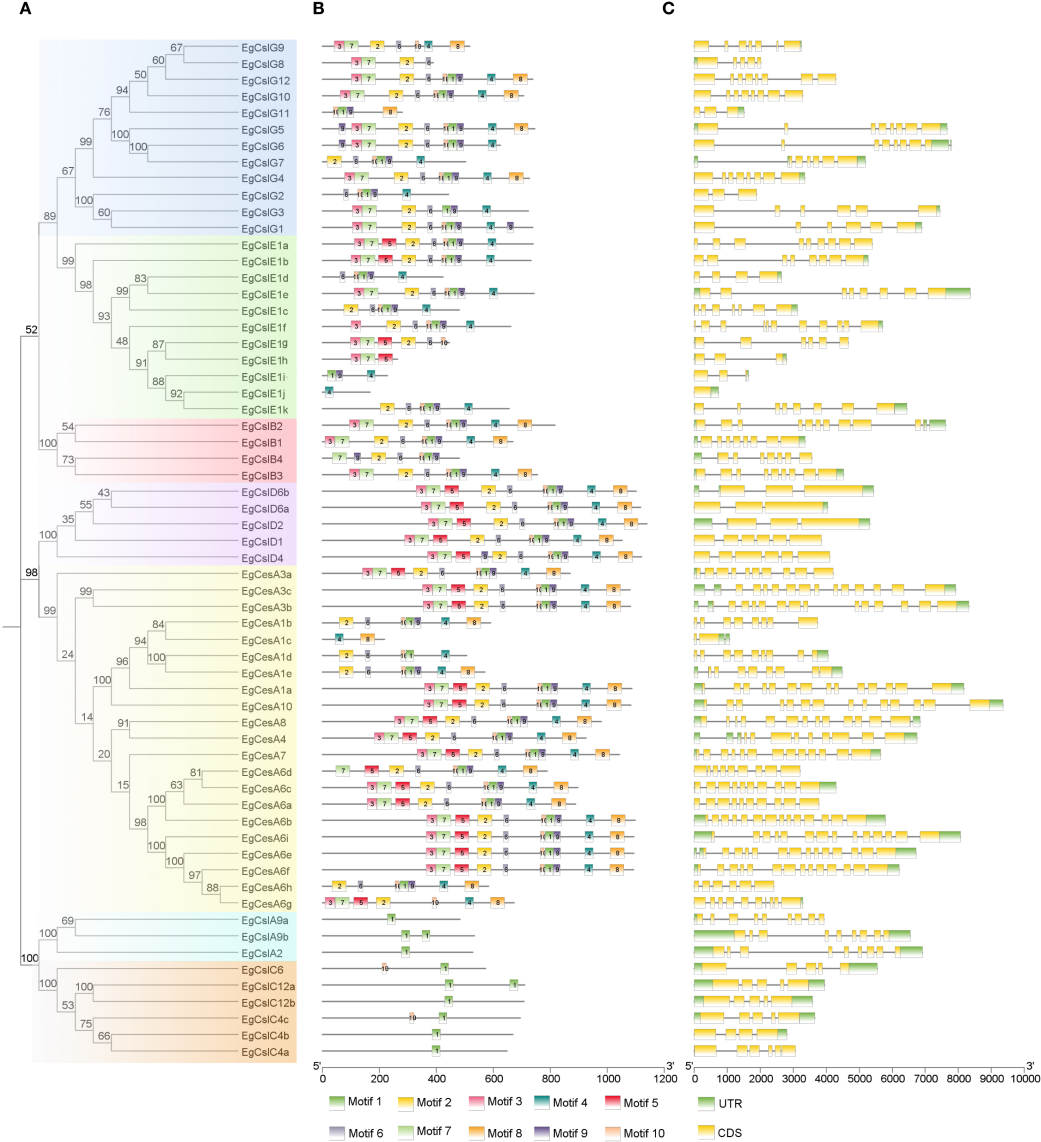
Predicted phylogeny, conserved amino acid motifs, and gene structure of EgCesA/Csls proteins. **(A)** Rooted maximum likelihood phylogeny of EgCesA/Csls proteins showing subfamily classification, with five different colors representing five subfamilies. **(B)** In *E*. *grandis*, the motif compositions of CesA/Csl proteins are represented by distinct colors, numbered from motif 1 to motif 10. **(C)** The intron-exon map illustrates the structural composition of the genes, with yellow regions representing the coding sequence (CDS) and green regions representing the untranslated regions (UTR). The relative positions are proportionally depicted at the bottom of the figure based on a kilobase scale.

Based on the characteristics of conserved protein motifs, the 62 *EgCesA/Csls* proteins were classified into five categories in the phylogenetic tree (**Figure 2A**). The conserved motifs of these 62 EgCesA/Csls proteins were visualized using the MEME online program (**Figure 2B**). There were relatively conserved domains at the amino terminal of EgCesA/Csls proteins. Conservative motif analysis showed that all *EgCesA* subfamily members contained Motif 4 and Motif 8, while *EgCslD* subfamily members contain all these motifs. All *EgCslA* and *EgCslC* subfamily members contain Motif1, and all *EgCslB* subfamily members contained Motif1, 2, 7, 9 and 10, but without Motif5; Motif4 were presented in *EgCslE* subfamily members except *EgCslE1h*, *EgCslE1g*. Additionally, the exon-intron distribution patterns of the *EgCesA/Csls* genes were investigated. As shown in **Figure 2C**, we found that number of exons ranged from 3 to 14. Furthermore, there was variability in the number of exons across different subfamily. For example, only 3–6 exons were presented in *EgCslD* subfamily, whereas more than 10 exons were existed in most of the members in *EgCesAs*.

### 3.4 Chromosomal localization and collinearity analysis of *EgCesA/Csls* gene family members

**Figure 3:**
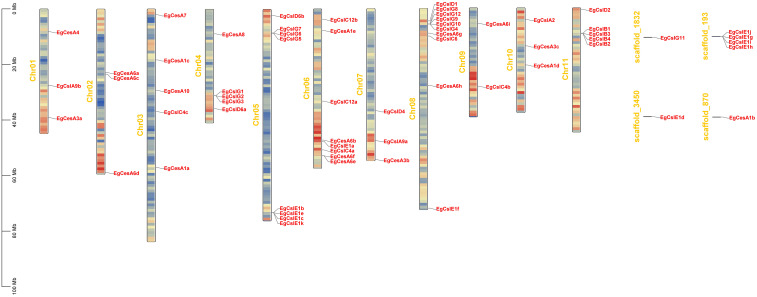
Chromosomal locations of *EgCesA/Csls* genes. Chromosome numbers are displayed in the middle of each chromosome. The scale bars indicate length in megabases (Mb). The red regions on each chromosome represent high gene density, while the blue regions indicate low gene density.

Based on the annotation, 55 genes were distributed across 11 chromosomes, while another 7 genes were located on scaffolds (**Figure 3**). Chromosomes 5, 6 and 8 contained a higher number of genes, ranging from 8 to 10 genes each. In contrast, chromosomes 1, 7, 9, and 10 harbored relatively few genes, with only 2 to 3 genes each. These 62 genes exhibited a diverse distribution pattern, including clustered and isolated distributions. 7 gene clusters (*EgCslG1*/*EgCslG2*/*EgCslG3*, *EgCslG5*/*EgCslG6*/*EgCslG7*, *EgCslE1b*/*EgCslE1e*/*EgCslE1c*/*EgCslE1k*, *EgCesA6f*/*EgCesA6e*, *EgCslG8*/*EgCslG12*/*EgCslG9*/*EgCslG10*/*EgCslG4*, *EgCslB1*/*EgCslB3*/*EgCslB4*/*EgCslB2*, *EgCslE1i*/*EgCslE1g*/*EgCslE1j/EgCslE1h*) were characterized as tandem repeat gene pairs, located on chromosomes chr04, chr05, chr06, chr08, chr11 and scaffold-193.

**Figure 4:**
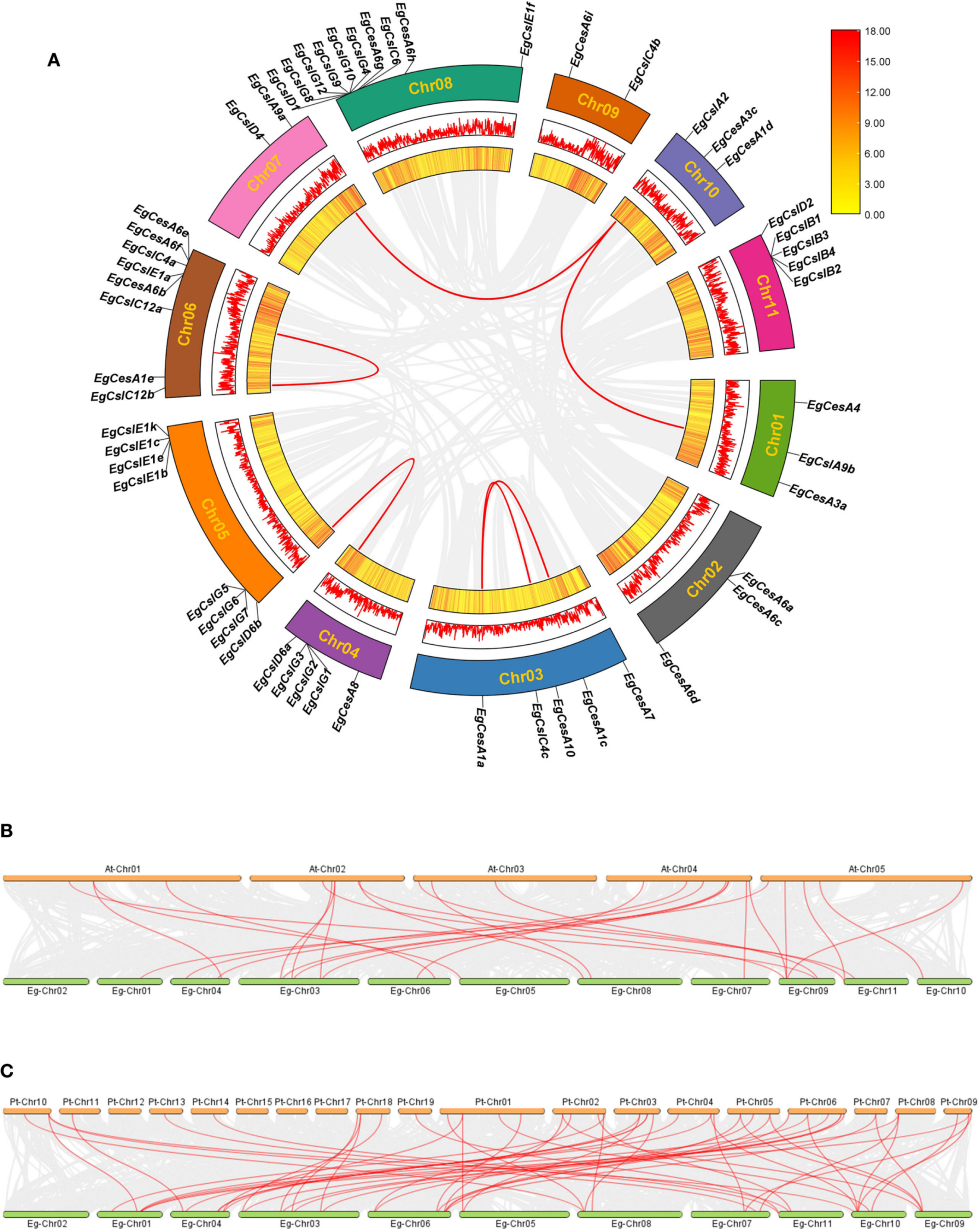
Collinearity relationship of *CesA/Csls* family members of *E. grandis*, *A*. *thaliana*, and *P. trichocarpa*. Intraspecific collinearity relationship of *CesA/Csls* family members of *E*. *grandis* **(A)**. The two rings from inside to outside represent gene densities, presented as HeatMap and Line, respectively, and the rectangle in the upper right corner represents the gene density scale. The outermost ring represents the chromosome position. The gray line indicates all collinearity blocks in the *E*. *grandis* genome, and the red line indicates the segmental repeats of the *EgCesA/Csls* genes. Interspecies collinearity relationship among E. grandis and *A*. *thaliana* **(B)** and *E*. *grandis* and *P. trichocarpa* **(C)** Gray lines in the background indicated all synteny blocks in the genome, while the red lines indicated the duplication of *CesA/Csls* gene pairs.

Intraspecific and interspecific collinearity of *EgCesA/Csls* family gene events was considered a major driving force in evolution (Panchy et al., 2016). In addition to tandem duplication, fragment duplication events within the *EgCesA/Csls* gene family were also conducted. The intraspecific collinearity analysis identified 6 collinear pairs among the gene family (**Figure 4A**), including *EgCslA9b* and *EgCslA2*, *EgCesA10* and *EgCesA1a*, *EgCesA1c* and *EgCesA1a*, *EgCslD6a* and *EgCslD6b*, *EgCslC12b* and *EgCslC12a*, *EgCslA9a* and *EgCslA2*. Each pair of collinear genes was situated on different chromosomes and was associated with fragment replication events. Taken together, the analysis of gene duplication events suggests that fragment replication serves as the main driving force behind the expansion of the EgCesA/Csls gene family.

To further elucidate the phylogenetic mechanisms of the *EgCesA/Csls gene* family, comparative syntenic maps were constructed, integrating *E. grandis* with two representative species of *A. thaliana* and *P. trichocarpa* (**Figures 4B, C**). The results revealed 26 *CesA/Csls* orthologous gene pairs (9 blocks for *EgCesA* gene and 17 blocks for *EgCsl* gene) between *E. grandis* and *A. thaliana*. Additionally, 53 collinear gene pairs (19 blocks for EgCesA gene and 34 blocks for EgCsl gene) were identified between *E. grandis* and *P. trichocarpa*. This result suggested that the *CesA/Csls* gene families in these two species had undergone numerous chromosomal segment rearrangements, gene duplications, and loss events during their evolutionary events. Based on these, it was speculated that *P. trichocarpa* and *E. grandis* might have a closer phylogenetic relationship.

### 3.5 Analysis of *cis-*acting elements in the *EgCesA/Csls* gene family

**Figure 5:**
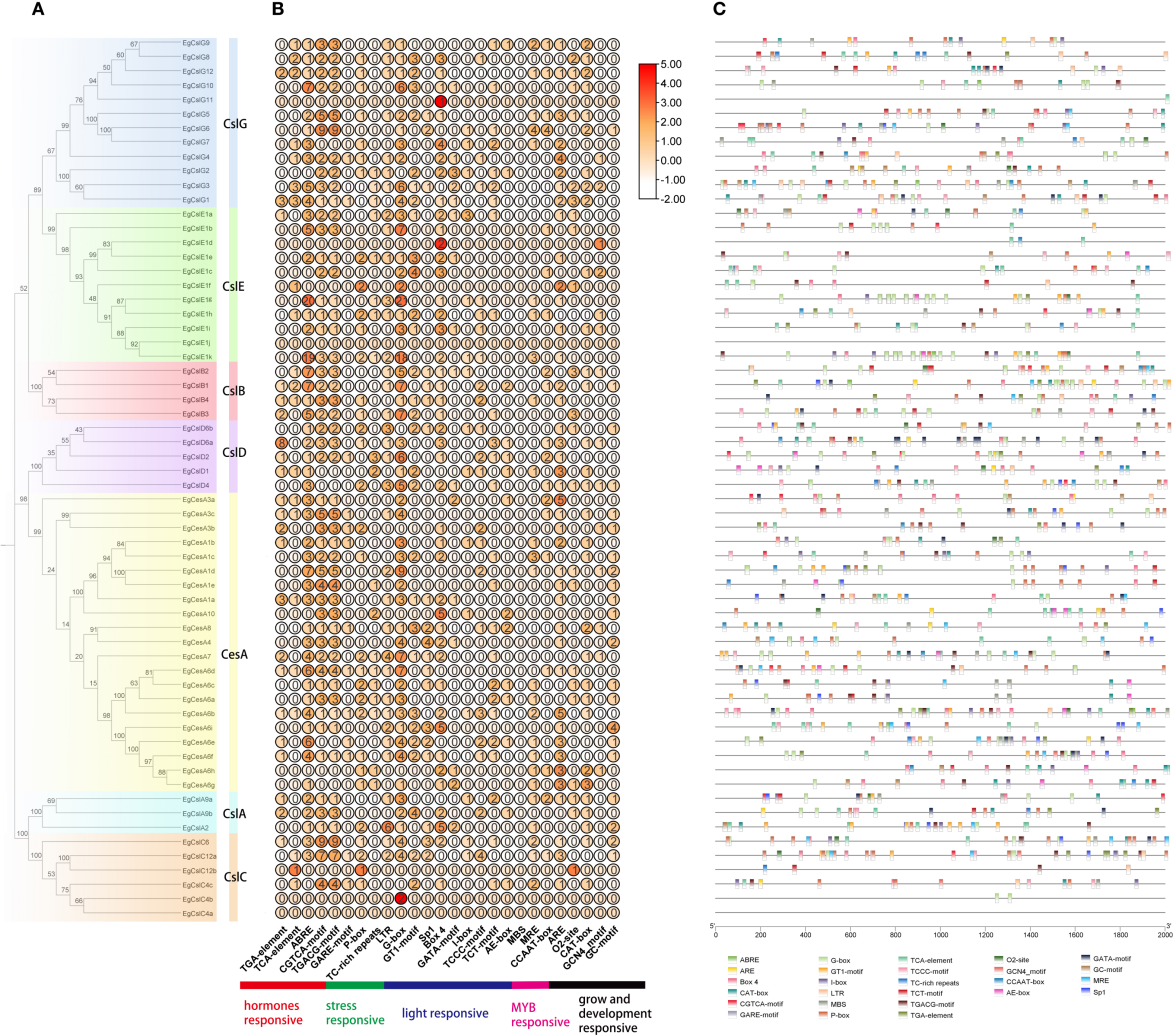
Analysis of *cis-*acting elements in the promoters of the *E*. *grandis EgCesA/Csls* gene family. **(A)** phylogenetic tree **(B)** A graph showing the count and expression of cis - acting elements according to response types **(C)** The 26 boxes on the right represent various *cis-*acting elements in the promoters of the *EgCesA/Csls* gene family.

Gene expression can be regulated through transcription factors (TFs) binding to *cis-*elements within their promoter regions (Wang et al., 2014). The *cis-*acting elements in the 2000 bp upstream promoter regions of *EgCesA/Csls* were analyzed alongside the phylogenetic tree (**Figure 5**). Among the 62 *EgCesA/Csls* genes, 26 response elements were selected and predicted. These elements were categorized into five types: hormone-responsive elements, MYB factor-responsive elements, light-responsive elements, growth and development-responsive elements, and stress-responsive elements (**Figure 5**). The analysis revealed that the *cis-*acting elements in the *EgCesA/Csls* gene family of *E. grandis* were diverse and abundant, with a single gene potentially involved in multiple physiological response processes. Taking the *EgCslG6* gene as an example, it contained various types of *cis-*acting elements, such as light responsive elements, hormone responsive elements, low-temperature responsive element and MYB factor-responsive elements. It was indicated that this gene played an important role in the stress resistance responses of *E. grandis*, especially in its response to low temperature stress. In addition, transcription factor binding site was predicted in the promoter of *EgCesA/Csls*. The result showed that the identification of 13 distinct transcription factor binding sites within the *EgCesA/Csls* promoter. Among these, the binding site of BBR-BPC (Barley B Recombinant/Basic Pentacysteine)transcription factor was the most abundant, found in the promoter regions of the majority of the CeslA and CeslC genes (**Supplementary Figure S1**). Besides, the Dof (DNA-binding with one finger) biding site was most prevalent in *EgCslE1c* promoter (with the number of 18), while the ERF (Ethylene Response Factor) binding sites was most commonly found in the promoter of *EgCesA4* (**Supplementary Figure S1**).

### 3.6 Expression of *EgCesA/Csls* in different tissues of *E. grandis*

**Figure 6:**
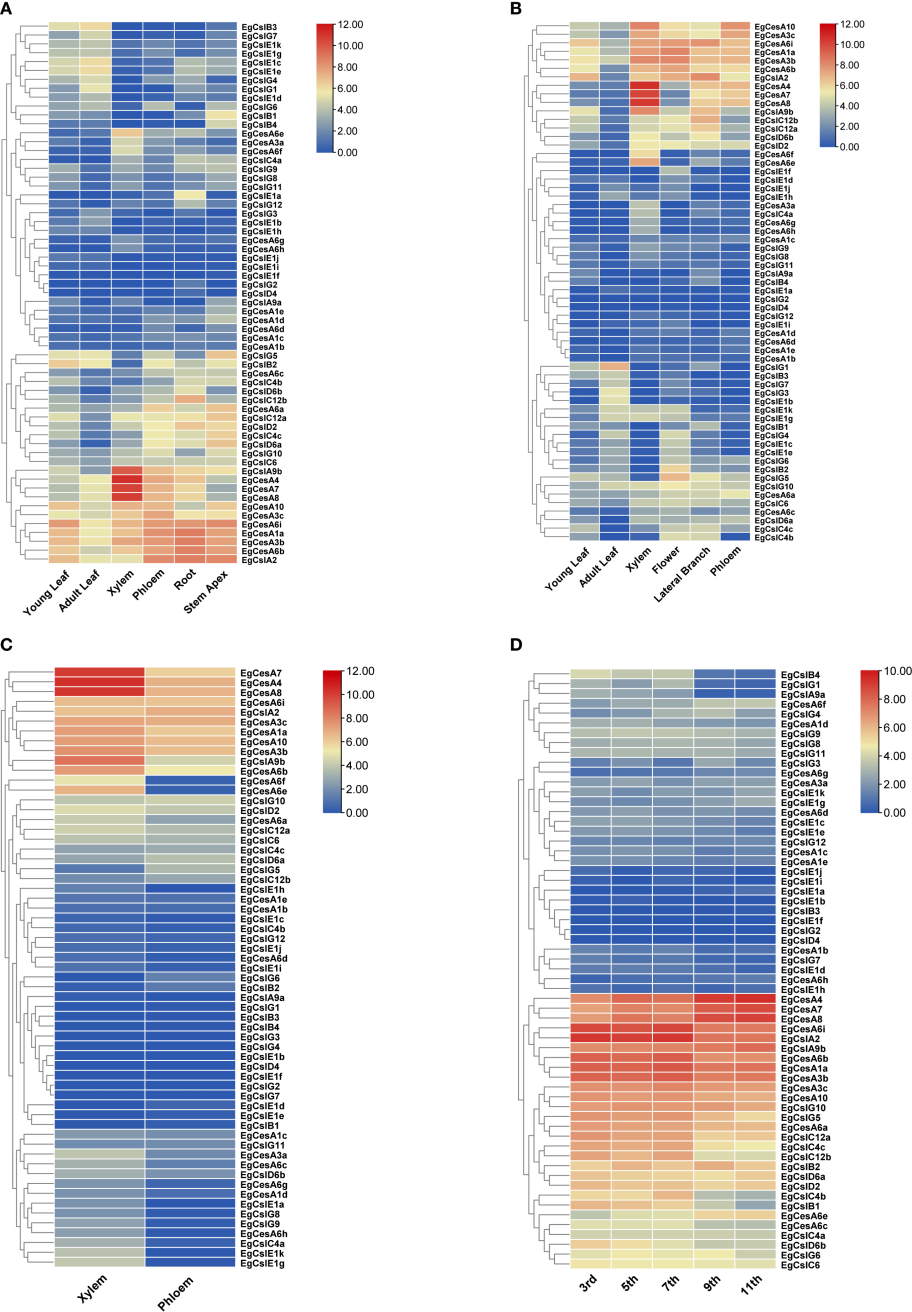
Heatmap of *EgCesA/Csls* gene expression in different tissues. Expression heatmaps of **(A)** young leaves, mature leaves, phloem, xylem, roots, and shoot tips of 6-month-old trees, **(B)** young leaves, mature leaves, xylem, flowers, lateral branches, and phloem of 3-year old trees, and **(C)** xylem and phloem of 6-year-old eucalyptus trees. **(D)** internodes 3, 5, 7, 9, and 11 of 6-month-old trees. Based on the RNA-seq data, the expression values are presented in the form of a heat map, with the color from blue to red corresponding to low to high expression.

The expression of different genes exhibited specificity across various tissue types (**Figure 6**). The result showed that the genes *EgCesA4*, *EgCesA7* and *EgCesA8* had highest expression levels in xylem across three distinct developmental stages: six months, three years, and six years (**Figures 6A-C**), suggesting that they would be crucial for xylem development. In addition, these genes were highly expressed in different internodes, particularly in the 7^th^ and 9^th^ internodes (**Figure 6D**), which are under secondary growth. The expression pattern of these three genes were consistent with developmental process of secondary wall formation. Besides, several *EgCesA* genes also exhibited high expression in phloem, root and stem apex, including *EgCesA1a*, *EgCesA3b*, *EgCesA3c*, *EgCesA6b*, and *EgCesA6i* (**Figures 6A, B**). However, low expression of the *EgCesA/Csl* genes in other subfamily were detected in xylem. These results indicated that the *EgCesA* subfamily play significant roles in xylem development.

### 3.7 Expression of *EgCesA/Csls* genes under abiotic stress and plant hormone treatments

**Figure 7:**
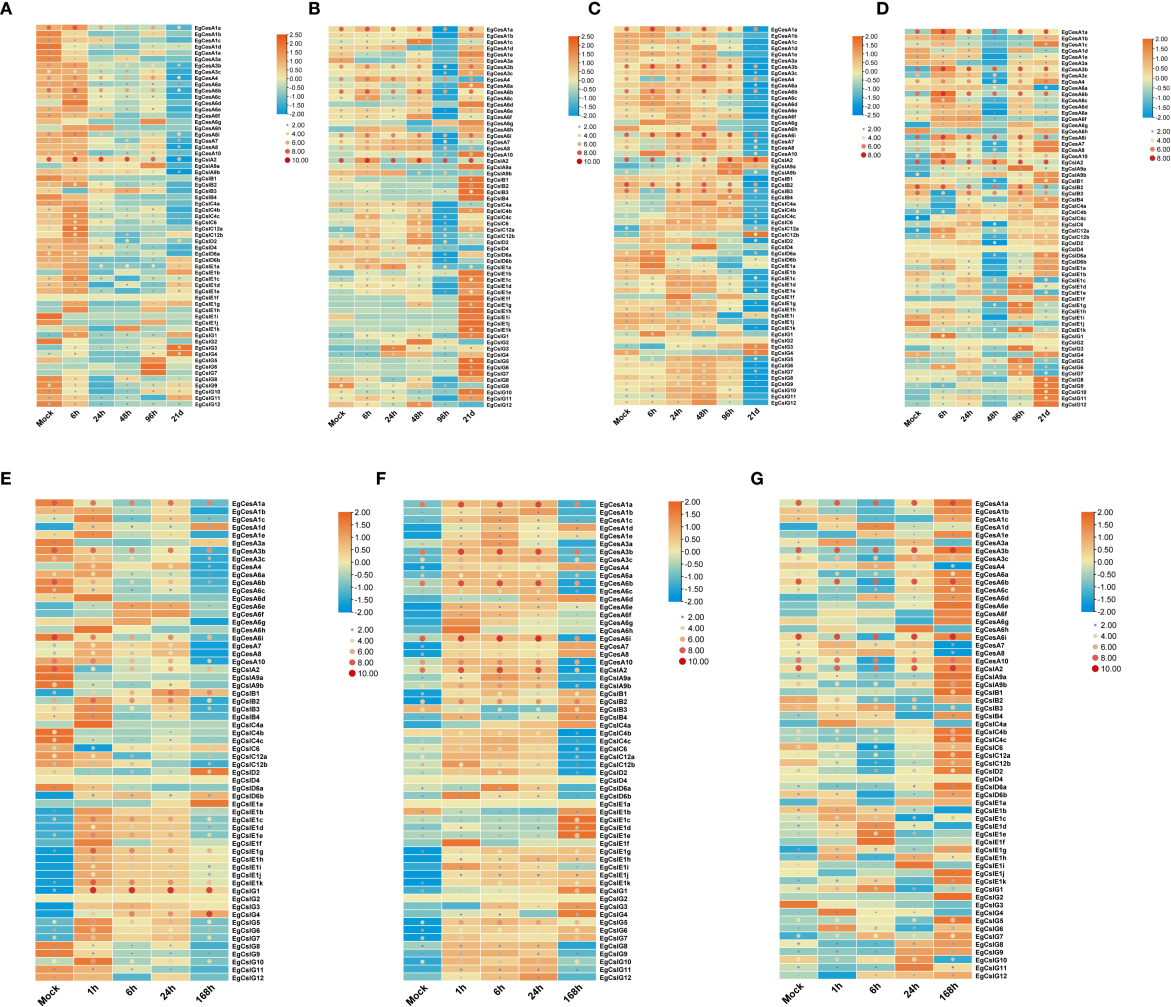
Gene expression of *EgCesA/Csls* genes under abiotic stress and hormone treatments. **(A-D)** Expression pattern of *EgCesA/Csls* genes under boron deficiency (a and c) and phosphorus deficiency treatment (b and d) after 0 h, 6 h, 24 h, 48 h, 96 h, and 21 **(D)** The root (a and b) and shoot (c and d) were used as samples. **(E-G)** Expression levels of *EgCesA/Csls* genes at 0 h, 1 h, 6 h, 24 h, and 168 h under salt stress **(E)**, JA **(F)** and SA treatment **(G)**. The heatmap was generated using TBtools-II based on the RNA-Seq data, with transformed data of log2(FPKM+1) value. Gene expression levels were represented by the color gradient, with orange indicating high expression and blue indicating low expression.Dot size and color represent EgCesA/Csls expression levels, with larger red dots indicating higher expression and smaller blue dots indicating lower expression. Gene cluster analysis was conducted on the gene expression levels by row.

To explore the expression dynamics of the *EgCesA/Csls* gene family under various abiotic stress conditions and phytohormone treatments, two-month-old *E. grandis* seedlings were subjected to a series of treatments. These included boron and phosphorus deficiency applied to both roots and stems, foliar application of salicylic acid (SA) and methyl jasmonate (MeJA), and exposure to salt stress. Gene expression responses were monitored to identify patterns of regulation under these stress conditions (**Figure 7**). The results revealed that *EgCesA1a* and *EgCslA2* was continued high expression across all treatments, including boron deficiency, phosphorus deficiency, salt stress, SA, and MeJA, suggesting its broad responsiveness to diverse environmental cues. Further analysis using row-normalized rectangle graphs showed clear temporal differences in gene expression patterns. For instance, under extended boron and phosphorus deficiency, *EgCslG3*, *EgCslG4* exhibited gradually increased expression levels over time, indicating a sustained response to nutrient deprivation. In contrast, *EgCesA1c*, *EgCesA4* showed only transient upregulation, peaking early during treatment and then declining, suggesting a short-term stress response. In terms of phytohormone treatments, *EgCesA3b*, *EgCesA6b*, *EgCesA1a* and *EgCslG1* genes showed significantly higher expression levels after SA and MeJA treatments, suggesting that they might play a key role in plant response to SA and MeJA. However, *EgCslD4* and *EgCslG2* genes were not expressed under salt stress, MeJA and SA treatment.

### 3.8 3D structure analysis of *EgCesA/Csls* gene family members

**Figure 8:**
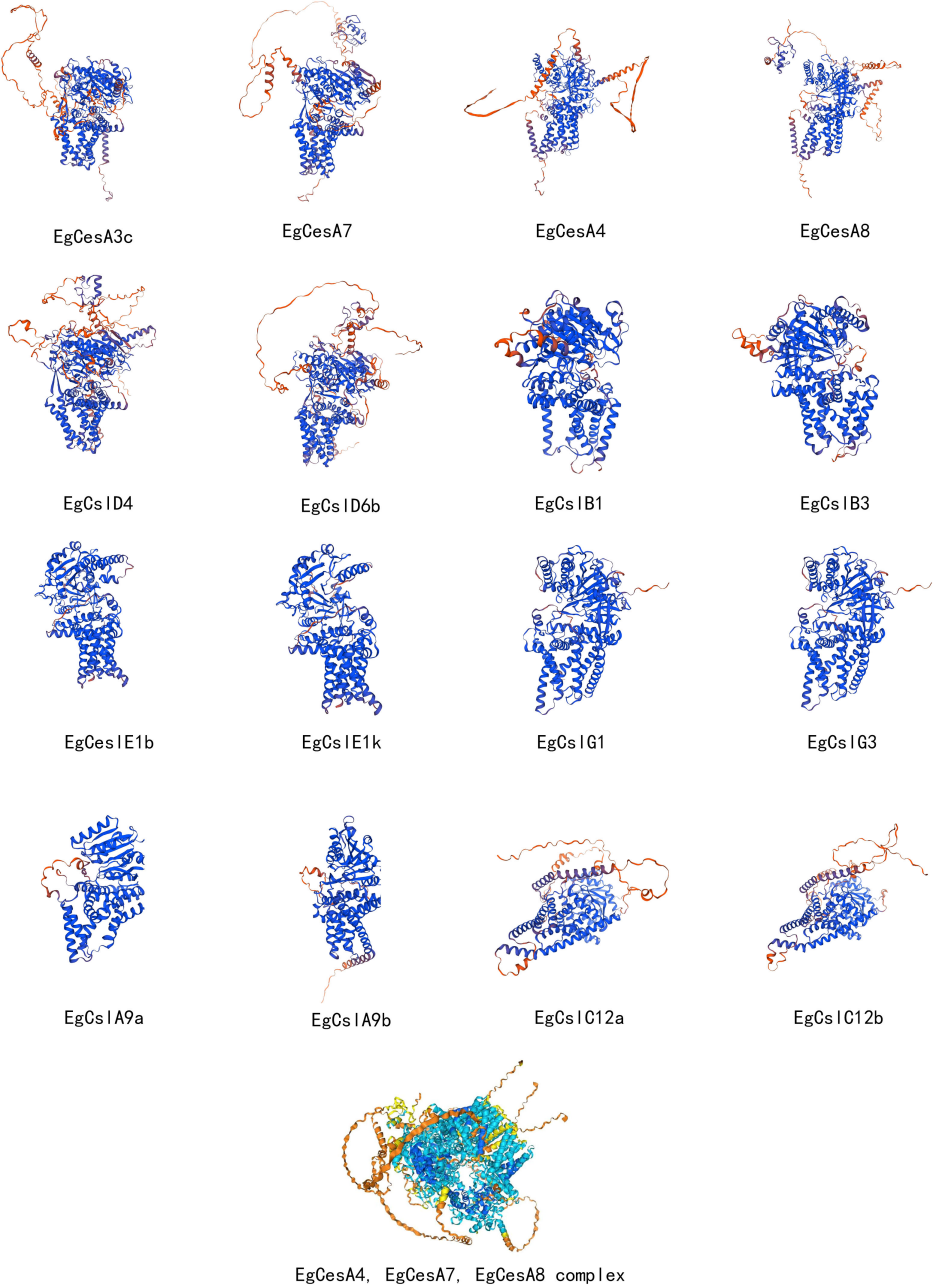
Three-dimensional structure analysis of members of the *EgCesA/Csls* gene family and *EgCesA4*, *EgCesA7*, *EgCesA8* complex. In the visualization of the *EgCesA4*, *EgCesA7*, and *EgCesA8* complexes, different colors indicate different binding strengths: dark blue indicates very high binding strength, light blue indicates confident binding strength, and yellow indicates low binding strength.

Homology modeling was conducted using Swiss-Model, and the models were evaluated with SAVES, successfully obtaining the three-dimensional structures of multiple representative *EgCesA/Csls* proteins (**Figure 8**). Two genes from each subfamily were selected for in-depth analysis of their three-dimensional structures, which revealed high similarity within the same subfamily, with minimal structural differences. Based on the prediction results, AlphaFold was used to resolve the CSC complex structure of *EgCesA4*, *EgCesA7*, and *EgCesA8*, which were involved in secondary cell wall formation. The analysis yielded an ipTM score of 0.61 and a pTM score of 0.66, indicating a high confidence in the interactions among these three genes (**Figure 8**).

## 4 Discussion

Glycosyltransferases (GTs) were a class of enzymes that catalyze the attachment of sugar molecules to specific receptors via glycosidic bonds, and they were widely present in various organisms including plants, animals, and microorganisms (Lairson et al., 2008). In plants, these enzymes were particularly important as they directly participate in carbon allocation and energy storage by converting monosaccharides (such as glucose) produced through photosynthesis into disaccharides, oligosaccharides, and polysaccharides (Keegstra and Raikhel, 2001). In addition, glycosyltransferases drove the synthesis of plant cell wall polysaccharides (such as cellulose and hemicellulose) and were involved in the glycosylation modification of glycoproteins, glycolipids, and secondary metabolites (such as flavonoids and alkaloids) (Bowles et al., 2006). These functions played a crucial role in maintaining the structural integrity of plant cell walls, signal transduction, and stress resistance (Zhao et al., 2022). According to the classification of the Carbohydrate-Active enZymes Database (CAZy), plant glycosyltransferases had formed highly diversified gene families (such as the GT2 family), with members exhibiting significant differentiation in substrate specificity and function (Lombard et al., 2014).

The *CesA* gene family was closely related to the biosynthesis of plant cellulose. During cell wall formation, the proteins encoded by *CesA* genes catalyzed the polymerization of glucose molecules to form cellulose microfibrils, which were crucial for maintaining the strength and structural stability of the cell wall (Zhang et al., 2016). In different plants, there were multiple members of the *CesA* gene family, and they exhibited functional differences (Kumar and Turner, 2015). For example, in *A.thaliana*, different *CesA* genes played specific roles in the synthesis of primary and secondary wall cellulose (Carroll and Specht, 2011). The *Csl* gene family was involved in the synthesis of various cell wall polysaccharides and was closely linked to the synthesis of substances such as hemicellulose (Richmond and Somerville, 2000; Doblin et al., 2010). The *Csl* gene family comprised numerous members, each with distinct functions, and exhibited specific expression patterns in different tissues and organs during plant growth and development (Hazen et al., 2002; Yin et al., 2009). In *A.thaliana*, 10 *CesA* genes and 31 cellulose synthase-like (*Csl*) genes had been identified, and based on phylogenetic analysis, they were further classified into one *CesA* family and six *Csl* families (*CslA/B/C/D/E/G*) (Persson et al., 2007b). Based on the phylogenetic analysis, we identified 21 *EgCesA* genes and 41 *Csl* genes, which were further classified into one *CesA* family and six *Csl* families. The CesA family can be divided into two subclasses, with EgCesA1/3/6 and EgCesA4/7/8 serving as representative members. EgCesA1/3/6 is likely participated in the synthesis of primary wall fibers during cell elongation, with *EgCesA1a*/*3b*/6b/6i having higher expression levels in rapidly growing tissues such as the shoot apex, young leaves, and root tips. In contrast, *EgCesA4/7/8* plays a role in cellulose synthesis during the thickening of secondary wall at the lignification stage, with increased expression levels observed in mature xylem and fiber cells (**Figures 1**, **6**). These is consistent with that in Arabidopsis and poplar (Taylor et al., 2003; Desprez et al., 2007; Abbas et al., 2020), suggesting their conservation in these plant species. *CslD* genes (*CslD1*, *CslD6a* and *CslD3*) in *A. thaliana* were involved in the synthesis of mannose-containing polysaccharides (Liepman et al., 2005; Yin et al., 2011). The *CslD* genes were localized on the Golgi membrane, where they catalyzed the synthesis of 1,4-β-D-glucomannan by substrate GDP-glucose and GDP-mannose (Huang et al., 2018; Yang et al., 2020). In the *CslD* subfamily shown in **Figure 6**, *EgCslD2* is highly expressed in roots and also highly expressed in stem apex, suggesting that CslD proteins may be involved in cellulose synthesis during root hair and shoot growth. *Eg CslD4* shows low expression levels in all tissues; *Eg CslD6a and Eg CslD6b* show higher expression levels in roots and stem apex, with lower levels in other regions. The diverse expression patterns of these group members suggest that subfunctionalization of genes may have occurred during their evolutionary process.

A phylogenetic tree analysis was conducted on the *CesA/Csls* gene families of *E. grandis*, *P. trichocarpa*, *A. thaliana*, and *P. patens*. *P. patens* is a bryophyte that serves as an outgroup for calibrating phylogenetic time nodes, facilitating the identification of the evolutionary origin of *E. grandis*. *A. thaliana*, a model organism for eudicots, diverged early in evolutionary history, and its annotated genome facilitates the examination of gene family alterations in *E. grandis. P. trichocarpa*, a woody eudicot that diverged approximately 100 million years ago, serves as a reference for investigating wood formation and metabolic processes in *E. grandis*. Although both species exhibit rapid growth, they differ in their lignin composition. Analyzing genes such as *CesA* provides insights into the evolutionary trajectory of lignin biosynthesis. Clustering analysis revealed that *EgCesA3c* clustered in the same branch with *AtCesA3* from *A. thaliana*, which were involved in primary cell wall cellulose synthesis (Desprez et al., 2007). This suggested that these *E. grandis CesA* genes may be involved in the synthesis of primary cell wall cellulose. *EgCesA4*, *EgCesA7*, and *EgCesA8* were phylogenetically closer to *AtCesA4*, *AtCesA7*, and *AtCesA8* from *A.thaliana*, respectively, which were involved in secondary cell wall cellulose synthesis (Arioli et al., 1998). There were 10 *CesA* genes in *A. thaliana*, whereas 21 *CesA* genes were identified in *E. grandis*, and although there was a significant increase in number, it was still three genes that were involved in secondary cell wall synthesis (*EgCesA4*, *EgCesA7*, and *EgCesA8*), and they were significantly expressed in the xylem in the subsequent tissue expression analysis. Therefore, it was inferred that these genes played a core role in secondary cell wall cellulose synthesis. *P. patens* genes were mainly distributed within the *CesA, CslA, CslC* and *CslD* families and exhibited a clustered distribution. In contrast, the tree species *E. grandis* and *P. trichocarpa* were more evenly distributed across the phylogenetic tree. This might be attributed to the fact that *P. patens* was a lower plant with a relatively low level of differentiation and slow species evolution. In contrast, *P. trichocarpa* and *E. grandis* have undergone more pronounced differentiation during evolution, better adapting to environmental changes, which reflected the strong stress resistance and stability of these species.

The expression heatmap analysis based on RNA-Seq data revealed that out of the gene set examined, 61 genes exhibited expression activity, with *EgCslD1* being silent and showing no detectable expression signal. Among the expressed genes, *EgCesA4*, *EgCesA7*, and *EgCesA8* demonstrated significant tissue-specific and developmental stage-specific expression patterns. These three genes were highly expressed in the xylem tissue of 6-month-old plants, with higher expression than those in other tissues and developmental stages. Moreover, *EgCesA4*, *EgCesA7*, and *EgCesA8* also showed high expression levels in the 3rd, 5th, 7th, 9th, and 11th internodes of 6-month-old plants, as well as in the xylem tissue of 6-year-old plants. This expression pattern further confirms that *EgCesA4*, *EgCesA7*, and *EgCesA8* were involved in the process of secondary wall thickening, specifically the cellulose biosynthetic pathway during the lignification stage, playing a crucial regulatory role in the formation of plant secondary cell walls and the lignification process. Cellulose synthase constitutes a rose knot-like cellulose synthase complex (CSC) at the plasma membrane, and two types of CSCs are involved in coordinated cellulose biosynthesis in the secondary cell wall of *Populus*, forming a multilayered structure that differentiates the xylem cells of *Populus* that undergo secondary cell wall thickening (Xi et al., 2017). In the present study, the high expression of EgCesA4, EgCesA7, and EgCesA8 in xylem in *E. grandis* might also constitute a complex involved in secondary cell wall synthesis and thickening. As is typical of woody plants, CSC and other complexes play important roles in cellulose structural characterization and multilayer wall structure formation during cell wall thickening in *P. trichocarpa* and *E. grandis*. Protein interaction network analysis is beneficial for predicting the composition of the cellulose synthase complex and elucidating its biological functions. Therefore, further investigation of the protein interaction network is necessary to characterize cellulose synthase complexes (CSCs) and their roles in cell wall synthesis.

When comparing the three-dimensional (3D) structures of proteins from different subfamilies, significant differences were observed. The results demonstrated high structural conservation within subfamily members, exhibiting only marginal variations (**Figure 8**). These structural variations were primarily attributed to the varying lengths of α-helices, β-turns, and γ-irregular coils among the proteins. The differences in these secondary structural elements resulted in altered folding angles of the proteins in space, and these changes in spatial conformation were highly likely to constitute the structural basis for the distinct functions of these proteins. Understanding the relationships between structure and function in cellulose synthases (CesAs) has been significantly enhanced by the partial elucidation of the structure of the CesA8 protein derived from poplar (PDB Entry-6WLB) using cryo-electron microscopy (Purushotham et al., 2020). PttCesA8 protein is a ortholog of Arabidopsis AtCesA8 and cotton GhCesA1, all of which belong to the cross-species CesA8 clade that facilitates the synthesis of cellulose in secondary walls (Zou et al., 2018). We compare the structure of EgCESA8 with PttCESA8 and AtCESA8, and found high similarity in these proteins, supporting the accuracy of the analysis (**Supplementary Figure S2**). The multiple alignment sequence were further conducted, suggesting their high conservation in the plant-conserved region (PCR), the class-specific region (CSR) and the conserved aspartic acids (D) and the QXXRW motif for donor and acceptor binding (**Supplementary Figure S1**). However, the members of CslA and CslC lack the PCR and CSR regions (Daras et al., 2021), resulting in a low-level similarity with CesA in structure (**Figure 8**). The rice *fc17* mutant of the CesA4 protein, which is the ortholog of Arabidopsis CesA8 participated in secondary cell wall synthesis, contains an amino acid substitution (F426S) in the PCR region. The *fc17* mutation influenced plant growth and resulted in increased lodging resistance, and also exhibited increased biomass saccharification efficiency and a decrease in cellulose content, accompanied by a compensatory rise in hemicellulose and lignin levels (Li et al., 2018). FxVTxK motif, which lies near the catalytic domain, exist within a substrate gating loop. Various mutation in this region showed change of the cellulose production (Daras et al., 2021). Therefore, these observations suggest that the PCR and FxVTxK motifs could serve as potential targets for cell wall modification in biotechnological applications. The FxVTxK motif was also investigated in EgCESA8, PttCESA8 and AtCesA8 (**Supplementary Figure S1**), and showing the conservation in this region. Additional functional studies on the FxVTxK motif in EgCESA8 are essential for understanding its role in cellulose synthesis. Through the analysis of the CSC complex structure formed by the proteins encoded by the three genes *EgCesA4*, *EgCesA7*, and *EgCesA8*, it was found that there was a cavity structure within the complex. It was speculated that this cavity structure could specifically bind to the substrate uridine diphosphate glucose (UDP-glucose). Knockout mutants of secondary cell wall CesA, including *irx5-4* (*cesa4*), *irx3-7* (*cesa7*), and *irx1-7* (*cesa8*) with T-DNA insertions, display a range of phenotypic characteristics such as dark green leaves and inflorescence stems, collapsed xylem vessels, decreased plant height and cellulose content (Kumar et al., 2017). This complex CSC model provided more intuitive and compelling structural biological evidence for the involvement of *EgCesA4*, *EgCesA7* and *EgCesA8* in the cellulose biosynthetic pathway during the lignification stage, further elucidating the mechanism of action of these three genes in this biological process at the molecular level.

*Cis-*regulatory elements analysis provides crucial information for transcriptional regulation of *CslC/CslA* gene (**Supplementary Table S2**). The majority of these gene promoters included response elements for hormones and abiotic stress (**Figure 5**). Specifically, *EgCesA1a* and *EgCslA2* exhibited consistently high expression levels across various treatments (**Figure 7**), which aligns with the presence of numerous *cis*-acting elements associated with hormone and stress responses (**Figure 5**). In addition, the high expression of these genes may also result from the combined effects of cross-talk among multiple signaling pathways (Wang et al., 2020), protein multifunctionality (Liu et al., 2017), and species-specific adaptive evolution (Wei et al., 2025), reflecting a strategy of “module reuse” by which plants cope with complex environmental stresses within a limited genome. Further functional investigation of these EgCesA/Csls by overexpression or knockout of these genes may help to elucidate their function in response to different stress conditions. Among the *cis*-acting elements identified in *EgCesA/Csls* genes promoters, MYB transcription factors related elements were widely distributed and presented in over half of the identified *EgCesA/Csls* (**Figure 5**). It was well-known the *MYB* transcription factors were highly expressed and widely involved in various stress responses, including drought and light responses, thereby regulating plant stress resistance, growth and development, and metabolic processes. In addition, MYB transcription factors could bind to the AC-box to regulate lignin biosynthesis genes (Xiao et al., 2021). Then they could directly promote secondary cell wall biosynthesis by regulating lignin, hemicellulose and cellulose pathway genes. Therefore, Data analysis from the heatmap showed that many members of this family were significantly expressed in the xylem, with lower expression in other tissues such as leaves, further highlighting their impact on cell wall formation. It has been found that two BBR-BPC transcription factors BPC1/BPC2 in *Arabidopsis* positively regulate plant β-1,4-galactan (a major component of pectin) accumulation (Yan et al., 2021). The binding sites of BBR-BPC were the most prevalent in the *EgCesA/Csls* promoters (**Supplementary Figure S2**), suggesting their potential roles in cell wall synthesis.

## 5 Conclusion

In this study, we identified 62 *EgCesA/Csls* genes containing cellulose synthase domains and classified them into seven subfamilies (*CesA*, *CslA*, *CslC*, *CslB*, *CslD*, *CslE*, and *CslG*). Furthermore, expression profile analysis showed that *CesA/Csls* genes exhibited different expression patterns in various tissues of *E. grandis*. Specifically, *EgCesA4*, *EgCesA7*, and *EgCesA8* were likely to be primarily involved in cellulose synthesis during the secondary wall thickening (lignification) stage according to their high expression in xylem. This finding lays a robust foundation for further investigation into the CesA/Csls-mediated molecular mechanisms underlying the growth and development process of *E. grandis.* Additionally, it offers a promising theoretical foundation and genetic resources for the enhancement of wood quality in *E. grandis*, as well as for improving biomass utilization efficiency through genetic engineering strategies.

## Data Availability

The data presented in the study are deposited in the Genome Sequence Archive (GSA) at the National Genomics Data Center repository, accession number PRJCA002468.

## References

[B1] Abbas M. Peszlen I. Shi R. Kim H. Katahira R. Kafle K.. (2020). Involvement of CesA4, CesA7-A/B and CesA8-A/B in secondary wall formation in Populus trichocarpa wood. Tree Physiol. 40, 73–89. doi: 10.1093/treephys/tpz020, PMID: 31211386

[B2] Arioli T. Peng L. Betzner A. S. Burn J. Wittke W. Herth W.. (1998). Molecular analysis of cellulose biosynthesis in *arabidopsis* . Science 279, 717–720. doi: 10.1126/science.279.5351.717, PMID: 9445479

[B3] Aspeborg H. Schrader J. Coutinho P. M. Stam M. Kallas Å. Djerbi S.. (2005). Carbohydrate-active enzymes involved in the secondary cell wall biogenesis in hybrid aspen. Plant Physiol. 137, 983–997. doi: 10.1104/pp.104.055087, PMID: 15734915 PMC1065399

[B4] Bowles D. Lim E.-K. Poppenberger B. Vaistij F. E. (2006). GLYCOSYLTRANSFERASES OF LIPOPHILIC SMALL MOLECULES. Annu. Rev. Plant Biol. 57, 567–597. doi: 10.1146/annurev.arplant.57.032905.105429, PMID: 16669774

[B5] Burton R. A. Gidley M. J. Fincher G. B. (2010). Heterogeneity in the chemistry, structure and function of plant cell walls. Nat. Chem. Biol. 6, 724–732. doi: 10.1038/nchembio.439, PMID: 20852610

[B6] Carroll A. Specht C. D. (2011). Understanding plant cellulose synthases through a comprehensive investigation of the cellulose synthase family sequences. Front. Plant Sci. 2. doi: 10.3389/fpls.2011.00005, PMID: 22629257 PMC3355508

[B7] Chen C. Chen H. Zhang Y. Thomas H. R. Frank M. H. He Y.. (2020). TBtools: an integrative toolkit developed for interactive analyses of big biological data. Mol. Plant 13, 1194–1202. doi: 10.1016/j.molp.2020.06.009, PMID: 32585190

[B8] Cocuron J.-C. Lerouxel O. Drakakaki G. Alonso A. P. Liepman A. H. Keegstra K.. (2007). A gene from the cellulose synthase-like C family encodes a β-1,4 glucan synthase. Proc. Natl. Acad. Sci. 104, 8550–8555. doi: 10.1073/pnas.0703133104, PMID: 17488821 PMC1895987

[B9] Daras G. Templalexis D. Avgeri F. Tsitsekian D. Karamanou K. Rigas S. (2021). Updating Insights into the Catalytic Domain Properties of Plant Cellulose synthase (CesA) and Cellulose synthase-like (Csl) Proteins. Molecules 26, 4335. doi: 10.3390/molecules26144335, PMID: 34299608 PMC8306620

[B10] Desprez T. Juraniec M. Crowell E. F. Jouy H. Pochylova Z. Parcy F.. (2007). Organization of cellulose synthase complexes involved in primary cell wall synthesis in *Arabidopsis thaliana* . Proc. Natl. Acad. Sci. 104, 15572–15577. doi: 10.1073/pnas.0706569104, PMID: 17878303 PMC2000492

[B11] Doblin M. S. Pettolino F. Bacic A. (2010). Plant cell walls: the skeleton of the plant world. Funct. Plant Biol. 37, 357. doi: 10.1071/FP09279

[B12] Doblin M. S. Pettolino F. A. Wilson S. M. Campbell R. Burton R. A. Fincher G. B.. (2009). A barley *cellulose synthase-like CSLH* gene mediates (1,3;1,4)-β- d -glucan synthesis in transgenic *Arabidopsis* . Proc. Natl. Acad. Sci. 106, 5996–6001. doi: 10.1073/pnas.0902019106, PMID: 19321749 PMC2667043

[B13] Fan C. Lyu M. Zeng B. He Q. Wang X. Lu M.. (2024). Profiling of the gene expression and alternative splicing landscapes of *Eucalyptus grandis* . Plant Cell Environ. 47, 1363–1378. doi: 10.1111/pce.14814, PMID: 38221855

[B14] Fang Y. Liao H. Wei Y. Yin J. Cha J. Liu X.. (2025). OsCDPK24 and OsCDPK28 phosphorylate heat shock factor OsHSFA4d to orchestrate abiotic and biotic stress responses in rice. Nat. Commun. 16 (1), 6485. doi: 10.1038/s41467-025-61827-6, PMID: 40659645 PMC12260056

[B15] Farrokhi N. Burton R. A. Brownfield L. Hrmova M. Wilson S. M. Bacic A.. (2006). Plant cell wall biosynthesis: genetic, biochemical and functional genomics approaches to the identification of key genes. Plant Biotechnol. J. 4, 145–167. doi: 10.1111/j.1467-7652.2005.00169.x, PMID: 17177793

[B16] Goubet F. Barton C. J. Mortimer J. C. Yu X. Zhang Z. Miles G. P.. (2009). Cell wall glucomannan in Arabidopsis is synthesised by CSLA glycosyltransferases, and influences the progression of embryogenesis. Plant J. 60, 527–538. doi: 10.1111/j.1365-313X.2009.03977.x, PMID: 19619156

[B17] Hazen S. P. Scott-Craig J. S. Walton J. D. (2002). Cellulose synthase-like genes of rice. Plant Physiol. 128, 336–340. doi: 10.1104/pp.010875, PMID: 11842136 PMC1540205

[B18] Huang Y.-P. He T.-B. Cuan X.-D. Wang X.-J. Hu J.-M. Sheng J. (2018). 1,4-β-d-Glucomannan from Dendrobium officinale Activates NF-кB via TLR4 to Regulate the Immune Response. Molecules 23, 2658. doi: 10.3390/molecules23102658, PMID: PMC6222441 30332800

[B19] Keegstra K. Raikhel N. (2001). Plant glycosyltransferases. Curr. Opin. Plant Biol. 4, 219–224. doi: 10.1016/S1369-5266(00)00164-3, PMID: 11312132

[B20] Kumar M. Atanassov I. Turner S. (2017). Functional analysis of cellulose synthase (CESA) protein class specificity. Plant Physiol. 173, 970–983. doi: 10.1104/pp.16.01642, PMID: 27923988 PMC5291044

[B21] Kumar M. Turner S. (2015). Plant cellulose synthesis: CESA proteins crossing kingdoms. Phytochemistry 112, 91–99. doi: 10.1016/j.phytochem.2014.07.009, PMID: 25104231

[B22] Lairson L. L. Henrissat B. Davies G. J. Withers S. G. (2008). Glycosyltransferases: structures, functions, and mechanisms. Annu. Rev. Biochem. 77, 521–555. doi: 10.1146/annurev.biochem.76.061005.092322, PMID: 18518825

[B23] Li G. Liu X. Liang Y. Zhang Y. Cheng X. Cai Y. (2020). Genome-wide characterization of the cellulose synthase gene superfamily in Pyrus bretschneideri and reveal its potential role in stone cell formation. Funct. Integr. Genomics 20, 723–738. doi: 10.1007/s10142-020-00747-8, PMID: 32770303

[B24] Li F. Liu S. Xu H. Xu Q. (2018). A novel FC17/CESA4 mutation causes increased biomass saccharification and lodging resistance by remodeling cell wall in rice. Biotechnol. Biofuels 11, 298. doi: 10.1186/s13068-018-1298-2, PMID: 30410573 PMC6211429

[B25] Li X. Tang C. Li X. Zhu X. Cai Y. Wang P.. (2022). Cellulose accumulation mediated by PbrCSLD5, a cellulose synthase-like protein, results in cessation of pollen tube growth in *Pyrus bretschneideri* . Physiol. Plant 174 (3), e13700. doi: 10.1111/ppl.13700, PMID: 35526262

[B26] Li L. Tang J. Wu A. Fan C. Li H. (2024). Genome-wide identification and functional analysis of the GUX gene family in eucalyptus grandis. Int. J. Mol. Sci. 25, 8199. doi: 10.3390/ijms25158199, PMID: 39125768 PMC11311485

[B27] Li Y. Yang T. Dai D. Hu Y. Guo X. Guo H. (2017). Evolution, gene expression profiling and 3D modeling of CSLD proteins in cotton. BMC Plant Biol. 17, 119. doi: 10.1186/s12870-017-1063-x, PMID: 28693426 PMC5504666

[B28] Liepman A. H. Wilkerson C. G. Keegstra K. (2005). Expression of cellulose synthase-like (*Csl*) genes in insect cells reveals that *CslA* family members encode mannan synthases. Proc. Natl. Acad. Sci. 102, 2221–2226. doi: 10.1073/pnas.0409179102, PMID: 15647349 PMC548565

[B29] Little A. Lahnstein J. Jeffery D. W. Khor S. F. Schwerdt J. G. Shirley N. J.. (2019). A novel (1,4)-β-linked glucoxylan is synthesized by members of the *cellulose synthase-like F* gene family in land plants. ACS Cent. Sci. 5, 73–84. doi: 10.1021/acscentsci.8b00568, PMID: 30693327 PMC6346400

[B30] Little A. Schwerdt J. G. Shirley N. J. Khor S. F. Neumann K. O’Donovan L. A.. (2018). Revised phylogeny of the *cellulose synthase* gene superfamily: insights into cell wall evolution. Plant Physiol. 177, 1124–1141. doi: 10.1104/pp.17.01718, PMID: 29780036 PMC6052982

[B31] Liu Y. Song Q. Li D. Yang X. Li D. (2017). Multifunctional roles of plant dehydrins in response to environmental stresses. Front. Plant Sci. 8. doi: 10.3389/fpls.2017.01018, PMID: 28649262 PMC5465263

[B32] Lombard V. Golaconda Ramulu H. Drula E. Coutinho P. M. Henrissat B. (2014). The carbohydrate-active enzymes database (CAZy) in 2013. Nucleic Acids Res. 42, D490–D495. doi: 10.1093/nar/gkt1178, PMID: 24270786 PMC3965031

[B33] Lou H. Tucker M. R. Shirley N. J. Lahnstein J. Yang X. Ma C.. (2022). The *cellulose synthase-like F3* (*CslF3*) gene mediates cell wall polysaccharide synthesis and affects root growth and differentiation in barley. Plant J. 110, 1681–1699. doi: 10.1111/tpj.15764, PMID: 35395116 PMC9324092

[B34] McFarlane H. E. Döring A. Persson S. (2014). The cell biology of cellulose synthesis. Annu. Rev. Plant Biol. 65, 69–94. doi: 10.1146/annurev-arplant-050213-040240, PMID: 24579997

[B35] Myburg A. A. Grattapaglia D. Tuskan G. A. Hellsten U. Hayes R. D. Grimwood J.. (2014). The genome of Eucalyptus grandis. Nature 510, 356–362. doi: 10.1038/nature13308, PMID: 24919147

[B36] Ouyang S. Zhu W. Hamilton J. Lin H. Campbell M. Childs K.. (2007). The TIGR Rice Genome Annotation Resource: improvements and new features. Nucleic Acids Res. 35, D883–D887. doi: 10.1093/nar/gkl976, PMID: 17145706 PMC1751532

[B37] Panchy N. Lehti-Shiu M. Shiu S.-H. (2016). Evolution of gene duplication in plants. Plant Physiol. 171, 2294–2316. doi: 10.1104/pp.16.00523, PMID: 27288366 PMC4972278

[B38] Park S. Szumlanski A. L. Gu F. Guo F. Nielsen E. (2011). A role for CSLD3 during cell-wall synthesis in apical plasma membranes of tip-growing root-hair cells. Nat. Cell Biol. 13, 973–980. doi: 10.1038/ncb2294, PMID: 21765420

[B39] Paterson A. H. Bowers J. E. Bruggmann R. Dubchak I. Grimwood J. Gundlach H.. (2009). The Sorghum bicolor genome and the diversification of grasses. Nature 457, 551–556. doi: 10.1038/nature07723, PMID: 19189423

[B40] Persson S. Caffall K. H. Freshour G. Hilley M. T. Bauer S. Poindexter P.. (2007a). The *Arabidopsis irregular xylem8* Mutant Is Deficient in Glucuronoxylan and Homogalacturonan, Which Are Essential for Secondary Cell Wall Integrity. Plant Cell 19, 237–255. doi: 10.1105/tpc.106.047720, PMID: 17237350 PMC1820957

[B41] Persson S. Paredez A. Carroll A. Palsdottir H. Doblin M. Poindexter P.. (2007b). Genetic evidence for three unique components in primary cell-wall cellulose synthase complexes in *Arabidopsis* . Proc. Natl. Acad. Sci. 104, 15566–15571. doi: 10.1073/pnas.0706592104, PMID: 17878302 PMC2000526

[B42] Polko J. K. Kieber J. J. (2019). The regulation of cellulose biosynthesis in plants. Plant Cell 31, 282–296. doi: 10.1105/tpc.18.00760, PMID: 30647077 PMC6447023

[B43] Purushotham P. Ho R. Zimmer J. (2020). Architecture of a catalytically active homotrimeric plant cellulose synthase complex. Science 369, 1089–1094. doi: 10.1126/science.abb2978, PMID: 32646917

[B44] Pydiura N. A. Bayer G. Y. Galinousky D. V. Yemets A. I. Pirko Y. V. Podvitski T. A.. (2015). Bioinformatic search and phylogenetic analysis of the cellulose synthase genes of flax (linum usitatissimum). Tsitol. Genet. 49, 3–12. doi: 10.3103/S0095452715050084, PMID: 26638491

[B45] Richmond T. A. Somerville C. R. (2000). The cellulose synthase superfamily. Plant Physiol. 124, 495–498. doi: 10.1104/pp.124.2.495, PMID: 11027699 PMC1539280

[B46] Sod B. Xu L. Liu Y. He F. Xu Y. Li M.. (2023). Genome-wide identification and expression analysis of the cesA/csl gene superfamily in alfalfa (Medicago sativa L.). Agriculture 13, 1658. doi: 10.3390/agriculture13091658

[B47] Takata N. Taniguchi T. (2015). Expression divergence of cellulose synthase (CesA) genes after a recent whole genome duplication event in Populus. Planta 241, 29–42. doi: 10.1007/s00425-014-2217-9, PMID: 25486888

[B48] Taylor N. G. Howells R. M. Huttly A. K. Vickers K. Turner S. R. (2003). Interactions among three distinct CesA proteins essential for cellulose synthesis. Proc. Natl. Acad. Sci. 100, 1450–1455. doi: 10.1073/pnas.0337628100, PMID: 12538856 PMC298793

[B49] Vega-Sánchez M. E. Verhertbruggen Y. Christensen U. Chen X. Sharma V. Varanasi P.. (2012). Loss of *cellulose synthase* - *like F6* function affects mixed-linkage glucan deposition, cell wall mechanical properties, and defense responses in vegetative tissues of rice. Plant Physiol. 159, 56–69. doi: 10.1104/pp.112.195495, PMID: 22388489 PMC3375985

[B50] Wang J. Li J. Lin W. Deng B. Lin L. Lv X.. (2022). Genome-wide identification and adaptive evolution of CesA/Csl superfamily among species with different life forms in Orchidaceae. Front. Plant Sci. 13. doi: 10.3389/fpls.2022.994679, PMID: 36247544 PMC9559377

[B51] Wang J. Song L. Gong X. Xu J. Li M. (2020). Functions of jasmonic acid in plant regulation and response to abiotic stress. Int. J. Mol. Sci. 21, 1446. doi: 10.3390/ijms21041446, PMID: 32093336 PMC7073113

[B52] Wang W. Wang L. Chen C. Xiong G. Tan X.-Y. Yang K.-Z.. (2011). Arabidopsis CSLD1 and CSLD4 are required for cellulose deposition and normal growth of pollen tubes. J. Exp. Bot. 62, 5161–5177. doi: 10.1093/jxb/err221, PMID: 21765162 PMC3193019

[B53] Wang W. Wu Y. Messing J. (2014). RNA-Seq transcriptome analysis of Spirodela dormancy without reproduction. BMC Genomics 15, 60. doi: 10.1186/1471-2164-15-60, PMID: 24456086 PMC3933069

[B54] Wei J. Han Y. Xu H. Deng L. Li L. Zhang S.. (2025). AAAP gene family evolution and transcriptional regulation in Eucalyptus grandis under nitrogen, phosphate and boron deficiencies. BMC Plant Biol. 25, 879. doi: 10.1186/s12870-025-06907-x, PMID: 40615974 PMC12232026

[B55] Welker C. Balasubramanian V. Petti C. Rai K. DeBolt S. Mendu V. (2015). Engineering plant biomass lignin content and composition for biofuels and bioproducts. Energies 8, 7654–7676. doi: 10.3390/en8087654

[B56] Xi W. Song D. Sun J. Shen J. Li L. (2017). Formation of wood secondary cell wall may involve two type cellulose synthase complexes in Populus. Plant Mol. Biol. 93, 419–429. doi: 10.1007/s11103-016-0570-8, PMID: 27987127

[B57] Xiao R. Zhang C. Guo X. Li H. Lu H. (2021). MYB transcription factors and its regulation in secondary cell wall formation and lignin biosynthesis during xylem development. Int. J. Mol. Sci. 22, 3560. doi: 10.3390/ijms22073560, PMID: 33808132 PMC8037110

[B58] Yan J. Liu Y. Yang L. He H. Huang Y. Fang L.. (2021). Cell wall β-1,4-galactan regulated by the BPC1/BPC2-GALS1 module aggravates salt sensitivity in Arabidopsis thaliana. Mol. Plant 14, 411–425. doi: 10.1016/j.molp.2020.11.023, PMID: 33276159

[B59] Yang J. Bak G. Burgin T. Barnes W. J. Mayes H. B. Peña M. J.. (2020). Biochemical and Genetic Analysis Identify CSLD3 as a beta-1,4-Glucan Synthase That Functions during Plant Cell Wall Synthesis. Plant Cell 32, 1749–1767. doi: 10.1105/tpc.19.00637, PMID: 32169960 PMC7203914

[B60] Yin Y. Huang J. Xu Y. (2009). The cellulose synthase superfamily in fully sequenced plants and algae. BMC Plant Biol. 9, 99. doi: 10.1186/1471-2229-9-99, PMID: 19646250 PMC3091534

[B61] Yin Y. Johns M. A. Cao H. Rupani M. (2014). A survey of plant and algal genomes and transcriptomes reveals new insights into the evolution and function of the cellulose synthase superfamily. BMC Genomics 15, 260. doi: 10.1186/1471-2164-15-260, PMID: 24708035 PMC4023592

[B62] Yin L. Verhertbruggen Y. Oikawa A. Manisseri C. Knierim B. Prak L.. (2011). The cooperative activities of CSLD2, CSLD3, and CSLD5 are required for normal arabidopsis development. Mol. Plant 4, 1024–1037. doi: 10.1093/mp/ssr026, PMID: 21471331

[B63] Zabotina O. A. Zhang N. Weerts R. (2021). Polysaccharide biosynthesis: glycosyltransferases and their complexes. Front. Plant Sci. 12. doi: 10.3389/fpls.2021.625307, PMID: 33679837 PMC7933479

[B64] Zhang B. Gao Y. Zhang L. Zhou Y. (2021). The plant cell wall: Biosynthesis, construction, and functions. J. Integr. Plant Biol. 63, 251–272. doi: 10.1111/jipb.13055, PMID: 33325153

[B65] Zhang T. Zheng Y. Cosgrove D. J. (2016). Spatial organization of cellulose microfibrils and matrix polysaccharides in primary plant cell walls as imaged by multichannel atomic force microscopy. Plant J. 85, 179–192. doi: 10.1111/tpj.13102, PMID: 26676644

[B66] Zhao H. Li Z. Wang Y. Wang J. Xiao M. Liu H.. (2022). Cellulose synthase-like protein OsCSLD4 plays an important role in the response of rice to salt stress by mediating abscisic acid biosynthesis to regulate osmotic stress tolerance. Plant Biotechnol. J. 20, 468–484. doi: 10.1111/pbi.13729, PMID: 34664356 PMC8882776

[B67] Zou X. Zhen Z. Ge Q. Fan S. Liu A. Gong W.. (2018). Genome-wide identification and analysis of the evolution and expression patterns of the cellulose synthase gene superfamily in Gossypium species. Gene 646, 28–38. doi: 10.1016/j.gene.2017.12.043, PMID: 29278771

