# Supplementary material for: Genome-wide identification and expression analysis of the CesA/Csls gene family in Eucalyptus Grandis
Source: Front Plant Sci. 2025 Oct 13;16:1624134. doi: 10.3389/fpls.2025.1624134 (PMC12554747; doi:10.3389/fpls.2025.1624134)
Supplement: Supplementary file 1 [file DataSheet1.pdf]

**Table S1 Mapping table of gene name and gene ID**

| Scientific name | Accession number | Phylogenetic tree name |
|---|---|---|
| *Arabidopsis thaliana* | NP_194967.1 | AtCesA1 |
| | NP_195645.1 | AtCesA2 |
| | NP_196136.1 | AtCesA3 |
| | NP_001332727.1 | AtCesA4 |
| | NP_196549.1 | AtCesA5 |
| | NP_201279.1 | AtCesA6 |
| | NP_197244.1 | AtCesA7 |
| | NP_567564.1 | AtCesA8 |
| | NP_179768.1 | AtCesA9 |
| | NP_001318288.1 | AtCesA10 |
| | NP_001319964.1 | AtCslA1 |
| | NP_197666.1 | AtCslA2 |
| | NP_173762.4 | AtCslA3 |
| | NP_565813.1 | AtCslA7 |
| | NP_195996.1 | AtCslA9 |
| | NP_173818.1 | AtCslA10 |
| | NP_001330941.1 | AtCslA11 |
| | NP_191159.2 | AtCslA14 |
| | NP_193077.2 | AtCslA15 |
| | NP_180820.2 | AtCslB1 |
| | NP_180821.1 | AtCslB2 |
| | NP_850190.1 | AtCslB3 |
| | NP_180813.1 | AtCslB4 |
| | NP_193264.3 | AtCslB5 |
| | NP_193267.1 | AtCslB6 |
| | NP_566835.1 | AtCslC4 |
| | NP_194887.1 | AtCslC5 |
| | NP_001326755.1 | AtCslC6 |
| | NP_001318283.1 | AtCslC8 |
| | NP_001328773.1 | AtCslC12 |
| | NP_001324935.1 | AtCslD1 |
| | NP_001318575.1 | AtCslD2 |
| | NP_186955.1 | AtCslD3 |
| | NP_195532.1 | AtCslD4 |
| | NP_171773.1 | AtCslD5 |
| | NP_001321681.1 | AtCslD6 |
| | NP_175981.2 | AtCslE1 |
| | NP_194132.3 | AtCslG1 |
| | NP_567692.2 | AtCslG2 |
| | NP_194130.3 | AtCslG3 |

| | | |
|---|---|---|
| Physcomitrium patens | XP_024400497.1 | PpCslD5j |
| | XP_024400497.1 | PpCslD5i |
| | XP_024394400.1 | PpCslD5h |
| | XP_024400347.1 | PpCslD5g |
| | XP_024376661.1 | PpCslD5f |
| | XP_024360967.1 | PpCslD5e |
| | XP_024378446.1 | PpCslD5d |
| | XP_024365235.1 | PpCslD5c |
| | XP_024365235.1 | PpCslD5b |
| | XP_024379454.1 | PpCslD5a |
| | XP_024367580.1 | PpCslC9 |
| | XP_024396723.1 | PpCslC8 |
| | XP_024385396.1 | PpCslC7 |
| | XP_024361017.1 | PpCslC6 |
| | XP_024361017.1 | PpCslC5 |
| | XP_024403147.1 | PpCslC4 |
| | XP_024358684.1 | PpCslC3 |
| | XP_024390769.1 | PpCslC2 |
| | XP_024398467.1 | PpCslC10 |
| | XP_024390774.1 | PpCslC1 |
| | XP_024396834.1 | PpCesA6m |
| | XP_024396834.1 | PpCesA6l |
| | XP_024396834.1 | PpCesA6k |
| | XP_024385240.1 | PpCesA6i |
| | XP_024384407.1 | PpCesA6h |
| | XP_024385240.1 | PpCesA6j |
| | XP_073392804.1 | PpCesA6g |
| | XP_024385029.1 | PpCesA6f |
| | XP_024363157.1 | PpCesA6e |
| | XP_024363157.1 | PpCesA6d |
| | XP_024363157.1 | PpCesA6c |
| | XP_024371386.1 | PpCesA6b |
| | XP_024382828.1 | PpCesA6a |
| | XP_024393618.1 | PpCslA3 |
| | XP_024400374.1 | PpCslA2 |
| | XP_024394673.1 | PpCslA1 |
| | XP_024371386.1 | PpCesA3c |
| | XP_024371386.1 | PpCesA3b |
| | XP_024371386.1 | PpCesA3a |
| Populus trichocarpa | XP_024466811.1 | PtCslG7 |
| | XP_024467111.1 | PtCslG6 |
| | XP_006385802.2 | PtCslG5 |
| | XP_006385802.2 | PtCslG4 |

| | | |
|---|---|---|
| *Populus trichocarpa* | XP_006385802.2 | PtCslG3 |
| | XP_002303664.2 | PtCslG2 |
| | XP_024452628.1 | PtCslG1 |
| | RQO91107.1 | PtCslE1d |
| | XP_002307850.1 | PtCslE1c |
| | XP_002307850.1 | PtCslE1b |
| | XP_024442552.2 | PtCslE1a |
| | KAI5605920.1 | PtCslD6c |
| | XP_002303441.1 | PtCslD6b |
| | XP_006368349.2 | PtCslD6a |
| | XP_002320989.2 | PtCslD5c |
| | XP_002320989.2 | PtCslD5b |
| | XP_002301494.3 | PtCslD5a |
| | XP_006384874.1 | PtCslD4d |
| | XP_006384874.1 | PtCslD4c |
| | XP_024464729.1 | PtCslD4b |
| | XP_024464729.1 | PtCslD4a |
| | XP_024446610.2 | PtCslD3c |
| | XP_024446608.2 | PtCslD3b |
| | XP_006376007.1 | PtCslD3a |
| | XP_024459313.2 | PtCslD1b |
| | XP_024453099.2 | PtCslD1a |
| | XP_002308730.1 | PtCslC8b |
| | XP_024445922.2 | PtCslC8a |
| | XP_002303072.1 | PtCslC6c |
| | XP_052302807.1 | PtCslC6b |
| | XP_002303072.1 | PtCslC6a |
| | XP_006384673.3 | PtCslC12a |
| | XP_002302382.1 | PtCslC12b |
| | XP_024440656.1 | PtCslB3 |
| | XP_024440656.1 | PtCslB2 |
| | XP_024450245.1 | PtCslB1 |
| | XP_024458242.2 | PtCslA9c |
| | XP_002315357.1 | PtCslA9b |
| | XP_002311972.2 | PtCslA9a |
| | XP_002312893.1 | PtCslA2a |
| | XP_002307301.1 | PtCslA2b |
| | XP_002316815.1 | PtCesA8d |
| | XP_002316815.1 | PtCesA8c |
| | XP_002316815.1 | PtCesA8b |
| | XP_024455193.1 | PtCesA8a |
| | XP_006381880.2 | PtCesA7c |
| | XP_006381880.2 | PtCesA7b |

| | | |
|---|---|---|
| *Populus trichocarpa* | XP_052304912.1 | PtCesA7a |
| | XP_002310628.1 | PtCesA6i |
| | XP_002310629.1 | PtCesA6h |
| | XP_002307145.1 | PtCesA6g |
| | XP_002307145.1 | PtCesA6f |
| | XP_002306707.1 | PtCesA6e |
| | XP_052306163.1 | PtCesA6d |
| | XP_024439473.2 | PtCesA6c |
| | XP_024439473.2 | PtCesA6b |
| | XP_006382504.2 | PtCesA6a |
| | XP_002301856.3 | PtCesA4b |
| | XP_002301856.3 | PtCesA4a |
| | XP_006369625.1 | PtCesA3e |
| | XP_006369625.1 | PtCesA3d |
| | XP_024463935.2 | PtCesA3c |
| | XP_006373780.1 | PtCesA3b |
| | XP_052309740.1 | PtCesA3a |
| | XP_024459034.1 | PtCesA10e |
| | XP_024459034.1 | PtCesA10d |
| | XP_024459034.1 | PtCesA10c |
| | XP_024459034.1 | PtCesA10b |
| | XP_002324291.2 | PtCesA10a |
