# Supplementary material for: Genome-wide identification and expression analysis of the CesA/Csls gene family in Eucalyptus Grandis
Source: Front Plant Sci. 2025 Oct 13;16:1624134. doi: 10.3389/fpls.2025.1624134 (PMC12554747; doi:10.3389/fpls.2025.1624134)
Supplement: Supplementary file 2 [file DataSheet2.pdf]

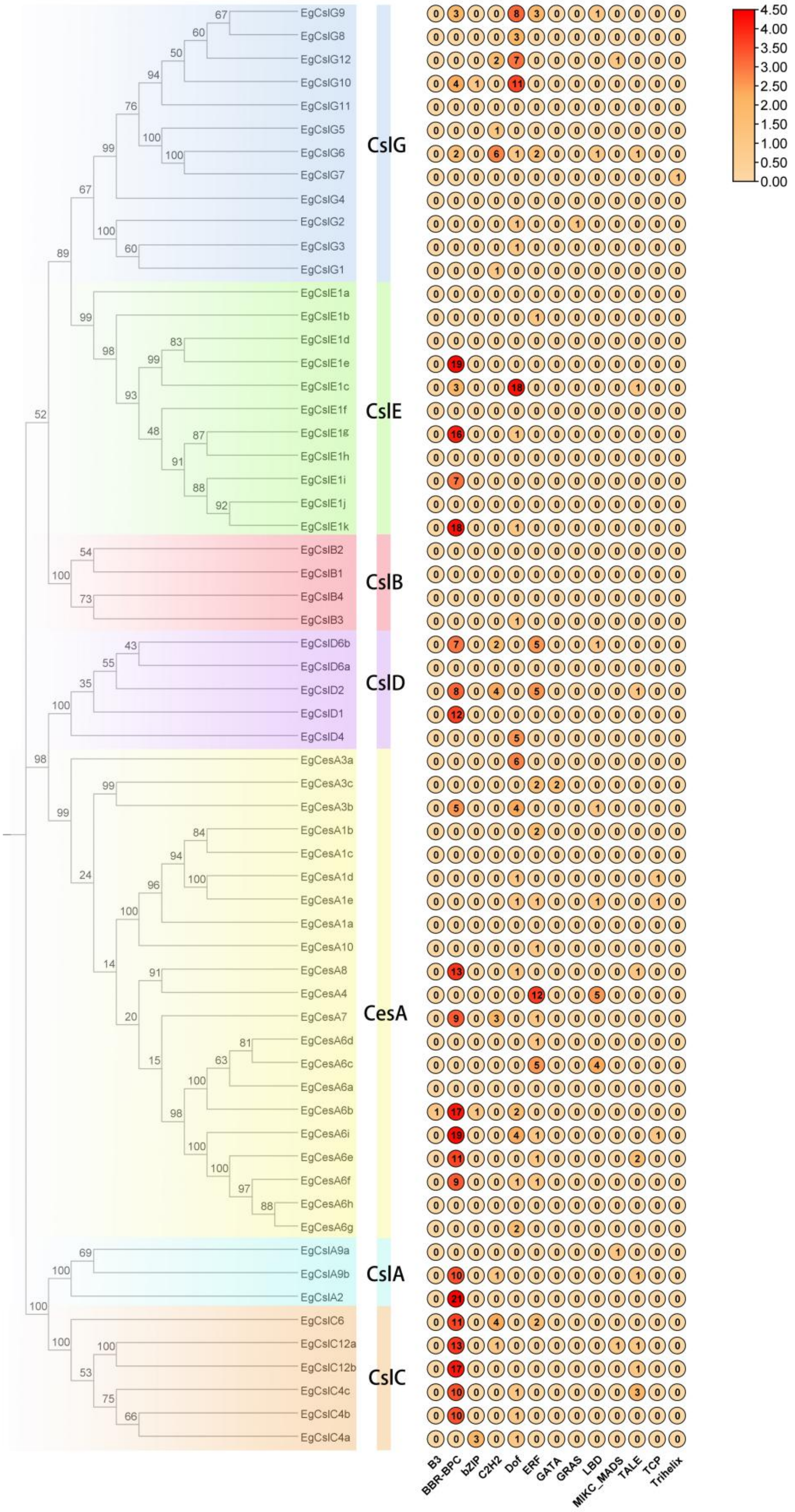

Figure S1 Prediction of transcription factor binding sites in *EgCesA/Csls* promoter. The numbers in the circle of right panel indicate the frequency of different transcription factors. The differently colored squares on the left panel represent distinct subfamilies.

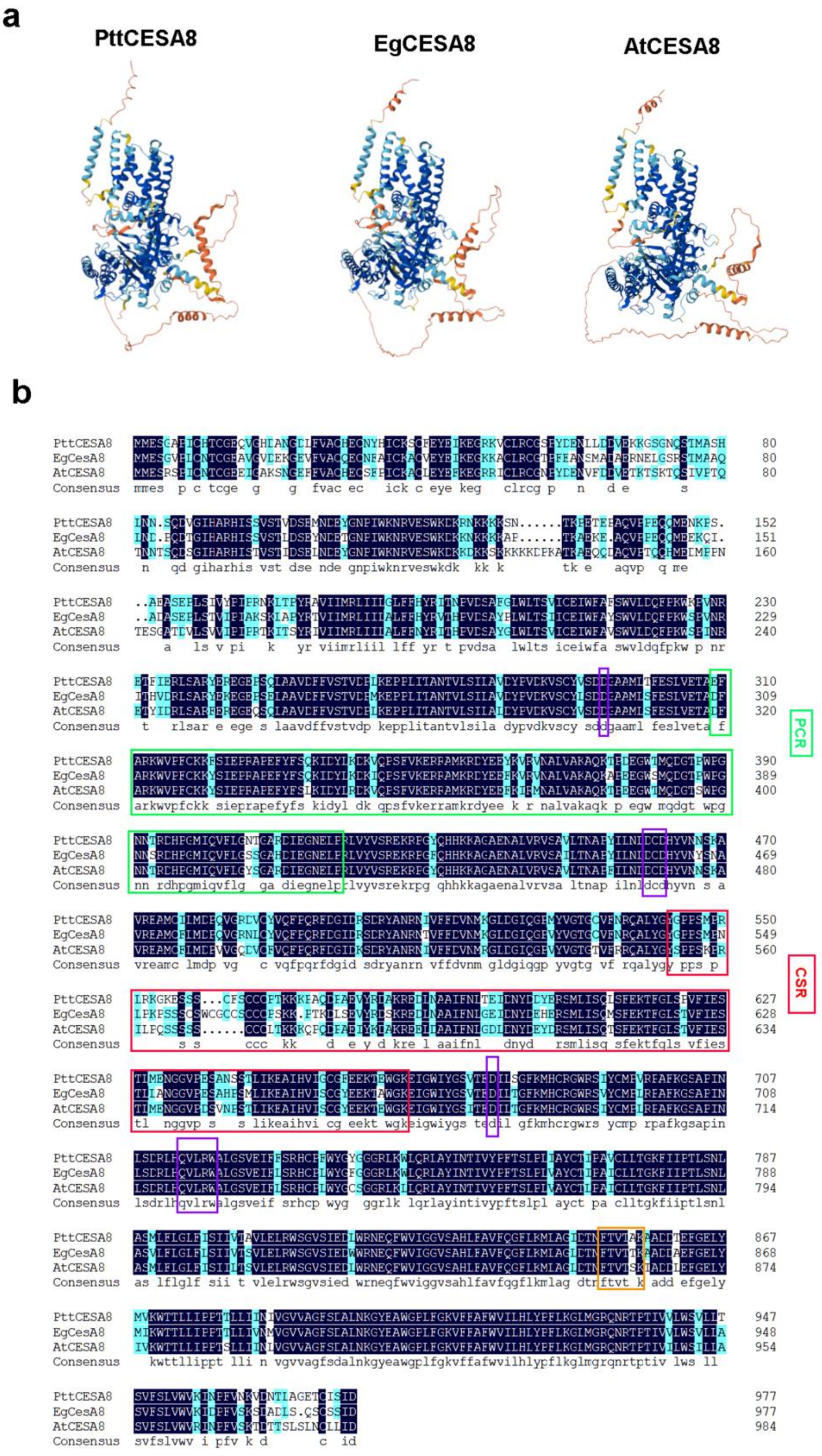

Figure S2 3D structure and multiple sequence alignment of EgCESA8, PttCESA8 (*Populus tremula x tremuloides*) and AtCESA8 (*Arabidopsis thaliana*). (a) 3D structure of EgCESA8, PttCESA8 and AtCESA8 proteins. The structure were predicted by AlphaFold. (b) Multiple sequence alignment of EgCESA8, PttCESA8 and AtCESA8. The plant-conserved region (PCR) and the class-specific region (CSR) are indicated by green and red box, respectively. Residues representing the conserved D,D,D, QxxRW motifs are boxed in purple. The orange box indicate the conserved FxVTxK motif.
